# Assessing Knowledge, Competence, and Performance Following Web-Based Education on Early Breast Cancer Management: Health Care Professional Questionnaire Study and Anonymized Patient Records Analysis

**DOI:** 10.2196/50931

**Published:** 2024-03-21

**Authors:** Michael Gnant, Khatijah Lim Abdullah, Frances Boyle, Chiun-Sheng Huang, Katie Bickford, Sola Neunie, Alexander Noble, Anne Nunn, Caroline Sproat, Nadia Harbeck, Carlos Barrios

**Affiliations:** 1 Comprehensive Cancer Center Medical University of Vienna Vienna Austria; 2 Department of Nursing School of Medical and Life Sciences Sunway University Selangor Malaysia; 3 Patricia Ritchie Centre for Cancer Care and Research University of Sydney Sydney Australia; 4 Department of Surgery National Taiwan University Hospital Taipei City Taiwan; 5 touch Independent Medical Education Limited Stockport United Kingdom; 6 Breast Center Department of Obstetrics & Gynecology and Comprehensive Cancer Center Munich University Hospital of Munich Ludwig Maximilian Munich Germany; 7 Latin American Cooperative Group and Oncoclínicas Group Hospital São Lucas da Pontifical Catholic University of Rio Grande do Sul Porto Alegre Brazil

**Keywords:** continuing medical education, early breast cancer, performance, risk stratification, shared decision-making

## Abstract

**Background:**

Web-based learning activities are key components of continuing medical education (CME) for health care professionals (HCPs). However, the published outcomes of web-based educational interventions for early breast cancer (EBC) are limited.

**Objective:**

This study aims to objectively assess knowledge, competence, and performance among HCPs following participation in 2 EBC-focused CME activities and to identify the remaining educational gaps.

**Methods:**

We developed 2 CME-accredited web-based educational activities addressing high-risk EBC, including integration of shared decision-making to optimize patient care (touchMDT) and stratification for early identification of high-risk patients and novel treatment strategies (touchPANEL DISCUSSION). Knowledge, competence, and performance were assessed before and after the activities against an expanded outcomes framework (levels 1-5) using self-reported questionnaires and an analysis of anonymized data extracted from patient records.

**Results:**

Six months after the launch of the activity, 7047 and 8989 HCP participants engaged with touchMDT and touchPANEL DISCUSSION, respectively. The overall satisfaction was 82% (a total score of 20.6 out of 25) for the touchMDT and 88% (a total score of 21.9 out of 25) for the touchPANEL DISCUSSION. For the evaluation of knowledge and competence (50 respondents before the activity and 50 learners after the activity), there was a significant increase in the mean number of correctly answered questions from pre- to postactivity (touchMDT: median 4.0, IQR 3.0-5.0 to median 5.5, IQR 4.0-7.0; mean 4.00, SD 1.39 to mean 5.30, SD 1.56 and touchPANEL DISCUSSION: median 4.0, IQR 4.0-5.0 to median 6.0, IQR 5.0-7.0; mean 4.32, SD 1.30 to mean 5.88, SD 1.49; both *P*<.001). A significant improvement in self-reported performance (50 respondents before the activity and 50 learners after the activity) was observed in a combined analysis of both activities (median 3.0, IQR 2.0-3.0 to median 4.0, IQR 3.0-5.0; mean 2.82, SD 1.08 to mean 4.16, SD 1.45; *P*<.001). Patient record analysis (50 respondents before the activity and 50 learners after the activity) showed that the HCPs used a range of measures to determine EBC recurrence risk and revealed no significant differences in adjuvant therapies used before and after the activity (*P*=.97 and *P*>.99 for Ki-67 <20% and Ki-67 ≥20% tumors, respectively). The remaining educational gaps included strategies for implementing shared decision-making in clinical practice and the use of genetic and biomarker testing to guide treatment selection.

**Conclusions:**

Brief, web-based CME activities on EBC were associated with an improvement in HCP knowledge, competence, and self-reported performance and can help identify unmet needs to inform the design of future CME activities.

## Introduction

### Clinical Decision-Making in High-Risk Early Breast Cancer

Clinical decision-making in cancer can be complex, requiring health care professionals (HCPs) to consider multiple clinical factors, patient preferences, and environmental factors [1-3]. A multidisciplinary team approach can improve the overall quality of decision-making [4-7], and there is evidence that shared decision-making (SDM) with patients can lead to better adherence to treatment and outcomes [1,8,9]. However, obstacles such as lack of resource availability and patient factors (eg, cultures and health literacy) have prevented the complete integration of SDM into cancer care [1,10-14]. HCPs need to understand these obstacles to deliver high-level care tailored to the individual.

The complexity of treating high-risk early breast cancer (EBC) has increased further with the emergence of complex new data on risk stratification and treatment. These include data on the prognostic and predictive value of biomarkers and the benefits of cyclin-dependent kinase 4 and 6 inhibitors, second-generation selective estrogen receptor antagonists and degraders, and protein kinase B (Akt) inhibitors [15-20]. Furthermore, the guidelines on the management of EBC have been updated recently to reflect recent data, which can be interpreted as influencing clinical practice [5,21-28]. Because of this rapid influx of complex information, HCPs who treat EBC are challenged to keep up with practice changes; consequently, it can take time for new and emerging data to be effectively integrated into practice, and patients with EBC may not receive optimal care [29-32].

### Effectiveness of Web-Based HCP Education in Breast Cancer

Effective continuing medical education (CME) programs are crucial to ensure that HCPs implement up-to-date, evidence-based practices for their patients. Although CME for HCPs has traditionally been delivered using face-to-face formats, web-based educational activities now offer a viable alternative approach [33]. To date, several studies have demonstrated the effectiveness of web-based HCP education for breast cancer [34-36]. However, gaps remain in the provision of independent web-based medical education programs for high-risk EBC treatment and effective SDM. Although a small number of programs have been developed in EBC [37-39], these have typically been nonaccredited, therefore not providing HCPs with continuing professional development credits for attendance; pharmaceutical company–led, therefore with the potential to be perceived as promotional; and restricted or limited in terms of access; therefore only accessible to those with the means to do so. To address these gaps and complement the currently available programs, accredited, independent, expert-led education available on free-to-access platforms is required to support the knowledge, competence, and performance of HCPs.

### Study Rationale, Objectives, and Aims

An expanded 7-level framework for assessing the outcomes of CME programs was developed by Moore et al [40] in 2009. This framework evaluates participation (level 1), satisfaction (level 2), knowledge (level 3), competence (level 4), performance (level 5), patient health status (level 6), and community health status (level 7). These levels are now widely used and included in many consensus documents and practice recommendations [41]. To date, published Moore’s level 5 [40] outcomes analyses in medical education have largely used subjective self-reported outcomes [42-44]. Although these can provide valuable insights, they can be open to bias, for example, recall bias or social desirability bias [45,46]. An analysis of anonymized patient records provides a complementary objective measure of performance that may enhance the understanding of physician behavior changes in practice.

In this study, we developed and implemented 2 faculty-led, CME-accredited web-based educational activities in high-risk EBC. The objectives of this study were to (1) measure changes in knowledge, competence, and self-reported performance after participation in the activities; (2) objectively evaluate changes in performance using anonymized patient records; and (3) identify the remaining educational gaps.

## Methods

### Study Design

This was a web-based study that analyzed the impact of 2 educational activities in high-risk EBC through HCP questionnaires and deidentified patient records. The target audience comprised oncologists (including breast cancer surgeons), oncology nurse specialists, pathologists, and radiologists from Asia, Brazil, and Europe (excluding the United Kingdom). Satisfaction, knowledge, competence, and performance were assessed according to levels 2 to 5 of the expanded framework for assessing the outcomes of CME programs by Moore et al [40] using self-reported questionnaires and anonymized data extracted from patient records. Questionnaire distribution and data collection were carried out by an independent third party (nuaxia Limited, United Kingdom).

### Ethical Considerations

All consents and ethical confirmations for this study were obtained before the completion of both outcome questionnaires. The personal data of HCPs and patients were anonymized. General Data Protection Regulation [47] consents were obtained in the European Union and North America for each questionnaire; adherence to additional requirements in local markets was required to be confirmed in line with European Pharmaceutical Market Research Association professional guidance [48]. For respondents to the patient records questionnaire, consents and ethical confirmations were obtained as part of the questionnaire in accordance with the British Healthcare Business Intelligence Association professional guidelines [49] to ensure patient confidentiality. As part of their written consent to participate in the questionnaires, the HCPs were required to agree to the statement, “As with all research, your identity and personal data are strictly confidential and will not be revealed without your explicit further consent. You have the right to withdraw your consent to participate in market research at any time.” Complete patient records were not obtained by touch Independent Medical Education (touchIME) or any third party; the HCPs were instructed to extract the required anonymized information into a questionnaire. Ethics approval was not applicable for this study because this analysis was not classified as “health research” according to the UK Research and Innovation definition that guides whether research requires Research Ethics Committee review and Health Research Authority approval [50,51]. The study was defined as “market research” per rule 1.1 of the European Pharmaceutical Market Research Association code of conduct, which states that market research does not require a Clinical Research Ethics Committee or independent review board approval [48].

### Educational Activities

Educational gaps and learning objectives were identified before the start of activity development in October 2021 by touchIME (a provider of IME for the global HCP community) through a review of the published literature. The expert faculty members further inputted into the educational gaps and learning objectives at the start of activity development. Expert faculty members with a background in breast cancer and SDM were identified by touchIME medical directors through searches of literature indexed in the PubMed database, congress websites, and web-based educational videos. Patient faculty were identified using social media searches across multiple channels. The faculty members (CB, FB, MG, NH, CSH and KLA) were blinded to the plans to assess participation and satisfaction with the education and to publish the outcomes. All the expert faculty (CB, FB, MG, NH, CSH and KLA) involved in the educational activities are authors of this manuscript.

A total of 2 web-based educational activities, touchMDT: “how can shared decision-making be successfully integrated to optimize care of patients with high-risk early breast cancer?” and touchPANEL DISCUSSION: “new horizons in high-risk HR+ HER2- EBC: Risk stratification for early identification and novel treatment strategies” were developed by touchIME in collaboration with the faculty members (CB, FB, MG, NH, CSH and KLA; complete details and learning objectives are included in Multimedia Appendix 1). The touchMDT activity comprised three 13- to 15-minute videos, providing 42 minutes of education in total, and the touchPANEL DISCUSSION activity comprised three 11- to 15-minute videos, providing 39 minutes of education in total. The activities were CME accredited by the Accreditation Council for Continuing Medical Education and the American Nurses Credentialing Center at the University of South Florida Health. Both activities were translated (video subtitles and downloadable slides) from English into Brazilian Portuguese, Chinese Mandarin, French, German, Italian, and Spanish by an independent third party (UnBabel, United States), and both activities were free to access for 1 year (the permissible lifetime of an accredited educational activity) on the touchONCOLOGY website from March 30, 2022, to March 30, 2023 (touchMDT) and from June 15, 2022, to June 15, 2023 (touchPANEL DISCUSSION). The 2 activities featured together on the host website, but participation in both activities was not mandatory.

Various communication channels were used to reach the target audience, including direct publicity emails to the touchONCOLOGY database within the first 12 weeks of the activity launch, with a further reminder at around 6 months; display banners on the touchONCOLOGY website; advertisements in the peer-reviewed journal touchREVIEWS in Oncology and Haematology; publicity via various relevant medical society partnerships; and social media partnerships on Facebook (Meta Platforms, Inc), LinkedIn (Microsoft Corp), and Twitter (rebranded as X; X Corp) targeted at the HCPs throughout the lifetime of the activity.

### Assessment of Educational Outcomes

#### Levels 1 and 2 (Participation and Satisfaction)

Levels 1 (participation) and 2 (satisfaction) were assessed separately for each activity (Multimedia Appendix 2). Level 1 included the number of HCPs who engaged in the web-based educational activities and the average time they spent viewing the videos, and it was the only assessment not evaluated using a questionnaire.

Level 2 (satisfaction) was assessed by scoring 5 statements of satisfaction (using a Likert scale) in a postactivity questionnaire.

#### Levels 3 to 4 and Level 5 (Knowledge, Competence, and Performance)

Levels 3 to 4 and level 5 were assessed using questionnaires completed by relevant respondents (HCPs completing the questionnaire before the activity) and learners (HCPs completing the questionnaire following the education). No pretesting was performed. The target audience was predefined by specialty (eg, oncologists [including breast cancer surgeons], oncology nurse specialists, pathologists, and radiologists) and by region (Brazil, France, Germany, Italy, and Spain). To avoid any pre-exposure bias and to obtain a statistically representative sample size, data were collected using an independent samples model for each activity. For levels 3 to 4, separate questionnaires were developed for each activity (each comprising 7 questions), whereas for level 5, a combined questionnaire was developed for both activities (3 questions each for touchMDT and touchPANEL DISCUSSION). All questions were developed by touchIME medical directors and writers and were approved for medical accuracy by the faculty. An overview of the topics and the complete questionnaires are provided in Multimedia Appendices 3 to 6.

For the touchPANEL DISCUSSION activity, changes in performance were also assessed through an evaluation of redacted patient records. The HCPs were sent a web-based questionnaire that captured data on (1) general patient demographics and history, (2) risk assessment and stratification to predict disease recurrence, and (3) treatment. Each HCP was requested to extract all relevant nonidentifiable information from a single anonymized patient record into the questionnaire.

### Self-Reported Confidence and Intention to Change Practice

As part of the questionnaires, respondents and learners were asked, “How confident are you in treating patients with breast cancer?” (mutually exclusive responses: “not confident,” “a little confident,” “somewhat confident,” “moderately confident,” and “extremely confident”) and “As a result of your participation in this session, will you make a change in your practice?” (mutually exclusive responses: “yes,” “uncertain—more education needed,” “uncertain—practical limitations,” “no—more education needed,” and “no—practical limitations”).

### Identification of Outstanding Educational Gaps

A total of 5 potential educational gaps were included in the levels 3 to 4 and level 5 questionnaires (Multimedia Appendix 7), and the learners were asked to rank them by importance. The results were analyzed using a single transferable vote system (Multimedia Appendix 8 [52]). In addition, questions that were answered incorrectly by ≥30% (15/50) of the learners in all postactivity questionnaires were identified as educational gaps. The 30% cutoff was determined by touchIME as an indicator of the requirement for further education based on analyses of data from previous outcomes.

### Fielding of the Questionnaires

Preactivity levels 3 to 4 questionnaires were fielded 1 to 2 weeks before launch, whereas postactivity levels 2 to 4 questionnaires were fielded immediately after launch to a different set of HCPs who had participated in the education. The questionnaires were distributed to a database of 20,420 HCPs and then “closed” once a prespecified number had responded (n=50).

The level 5 questionnaire and first patient records questionnaire were fielded 1 to 2 weeks before launch—to a different set of HCPs than those who answered the levels 3 to 4 questionnaires—and closed after completion by a prespecified number of respondents (n=50). A total of 26 weeks after launch, the postactivity level 5 questionnaire and second patient records questionnaire were distributed to the same set of HCPs who completed the level 5 questionnaire and patient records questionnaire before launch. Because of drop out over time, additional HCPs who were matched to the dropouts were surveyed postactivity, if needed.

The HCP participants’ specialties were all validated by nuaxia Limited against third-party data with the assistance of artificial intelligence. This comprised any combination of data from professional associations, hospitals, professional publications, and prescribing and insurance databases.

The participants were required to answer “yes” to the screening question “Do you treat patients with early breast cancer?” to be eligible to participate in the questionnaire study. The HCPs eligible to participate received an honorarium in the form of a digital reward, which could be redeemed via a number of reward partners. In accordance with industry regulations and professional body guidance, the honorarium received was limited to the fair market value for an HCP of that seniority.

### Statistical Analyses

Data were analyzed using SPSS Statistics software (version 28.0.1; IBM Corp). The sample size (50 respondents and 50 learners for each activity) was predetermined by the level of funding, and by applying a power analysis, we determined a resulting margin of error of 10% for both the touchMDT and touchPANEL DISCUSSION. Knowledge, competence, and self-reported outcomes were compared for the overall population using an independent samples 2-tailed *t* test. Data were subdivided by region and years of experience, and a 2-way ANOVA was used to assess the variation in results by these demographics. Individual questions were analyzed with a paired samples 2-tailed *t* test and a 1-way ANOVA, followed by a cluster analysis, to provide insights into the overall change in correct answers across the questions asked and the specific changes in all the answers given. Data on the number of questions answered correctly were measured as a continuous variable. At the individual question level, the answers were categorical. For performance, measured by the redacted patient records questionnaire, pre- and postactivity comparisons were made using paired and independent samples 2-tailed *t* tests, chi-square tests, and cluster analysis. These tests were used to assess patient history, use of risk assessment and stratification strategies, and patient treatment pathways.

## Results

### Assessment of Educational Outcomes

#### Levels 1 and 2 (Participation and Satisfaction)

By 6 months after launch, 7047 and 8989 participants had engaged with the touchMDT and touchPANEL DISCUSSION activities, respectively. The participants were predominantly oncologists, and the majority were based in Italy for the touchMDT and Brazil for the touchPANEL DISCUSSION activities (Multimedia Appendix 9). Overall satisfaction was 82% (a total score of 20.6 out of 25) for touchMDT and 88% (a total score of 21.9 out of 25) for touchPANEL DISCUSSION (Multimedia Appendix 10). Multichannel publicity reach and impact for the touchMDT and touchPANEL DISCUSSION activities are reported in Multimedia Appendix 11.

#### Levels 3 and 4 (Knowledge and Competence)

A total of 50 respondents and 50 learners were questioned to assess levels 3 to 4 outcomes (Table 1).

**Table 1:** Demographics of participating health care professionals (HCPs) for the touchMDT and touchPANEL DISCUSSION activities^a^.

Demographics of HCPs		Levels 2 to 4 questionnaire					Level 5 questionnaire	
		touchMDT		touchPANEL DISCUSSION		Both activities		
		Respondents	Learners	Respondents	Learners	Respondents		Learners
Participants, N		50	50	50	50	50		50
**Specialty, n (%)**								
	Oncologist	40 (80)	42 (84)	44 (88)	47 (94)	43 (86)		42 (84)
	Radiologist	0 (0)	0 (0)	6 (12)	3 (6)	4 (8)		3 (6)
	Pathologist	0 (0)	0 (0)	0 (0)	0 (0)	3 (6)		5 (10)
	Radiologist and pathologist	10 (20)	8 (16)	0 (0)	0 (0)	0 (0)		0 (0)
**Years of practice, n (%)**								
	<1 to 5	6 (12)	5 (10)	N/A^b^	N/A	N/A		N/A
	>5 to 20	29 (58)	25 (50)	N/A	N/A	N/A		N/A
	<10	N/A	N/A	10 (20)	11 (22)	N/A		N/A
	10 to 20	N/A	N/A	27 (54)	27 (54)	N/A		N/A
	0 to 20	N/A	N/A	N/A	N/A	30 (60)		32 (64)
	>20	15 (30)	20 (40)	13 (26)	12 (24)	20 (40)		18 (36)
**Country, n (%)**								
	Brazil	14 (28)	11 (22)	16 (32)	15 (30)	0 (0)		0 (0)
	France	5 (10)	9 (18)	5 (10)	5 (10)	7 (14)		7 (14)
	Germany	14 (28)	12 (24)	16 (32)	10 (20)	16 (32)		18 (36)
	Italy	8 (16)	9 (18)	7 (14)	10 (20)	13 (26)		11 (22)
	Spain	9 (18)	9 (18)	6 (12)	10 (20)	14 (28)		14 (28)

^a^Data were collected 6 months after the launch of the touchMDT and touchPANEL DISCUSSION activities on September 29, 2022, and November 21, 2022, respectively. The questionnaire response categories differed between questionnaires and activities, meaning that some options were not applicable. The respondents and learners were the HCPs who completed the pre- and postactivity questionnaires, respectively.

^b^N/A: not applicable.

**Figure 1:**
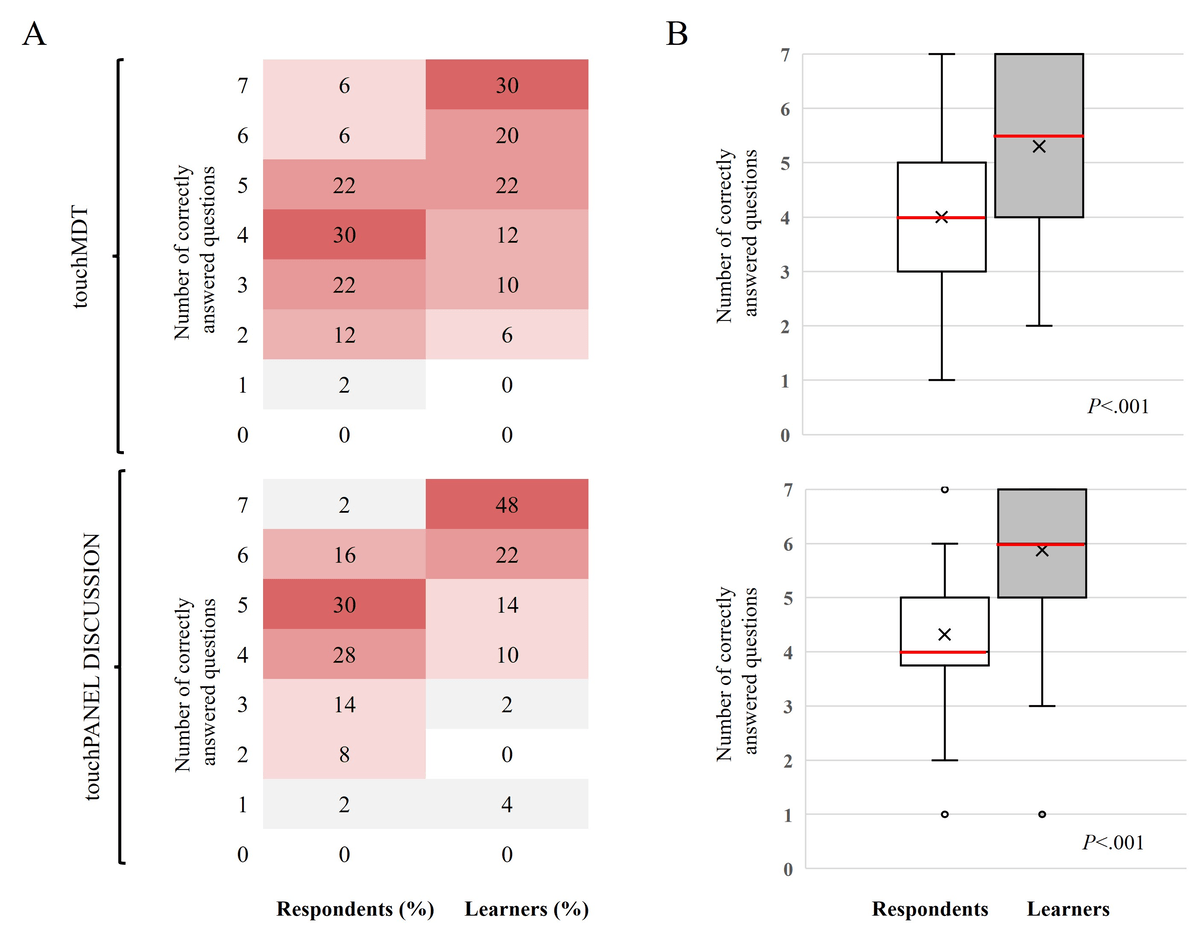
Summary of the number of correct responses for the levels 3 and 4 outcomes questionnaire before and after the launch of touchMDT and touchPANEL DISCUSSION. (A) Heat maps show the proportion of respondents (n=50) and learners (n=50) who answered a specific number of questions correctly, as displayed by colors ranging from white (the lowest proportion of respondents and learners) to dark red (the highest proportion of respondents and learners). (B) Box and whisker plots show the distribution of the number of questions correctly answered by all respondents and learners. In both plots, the horizontal red line within the box indicates the median, the “x” symbol represents the mean, the boxes indicate the IQR, and the vertical lines (whiskers) extend to the range of values, excluding outliers.

For the touchMDT and touchPANEL DISCUSSION activities, respectively, only 12% (6/50) and 18% (9/50) of the respondents answered at least 6 (86%) of the 7 questions correctly before the activity. This increased to 50% (25/50) and 70% (35/50) in the learners, respectively, after the activity (Figure 1). In Figure 1A, the heat maps show the proportion of respondents (N=50) and learners (n=50) who answered a specific number of questions correctly, as displayed by colors ranging from white (the lowest proportion of respondents and learners) to dark red (the highest proportion of respondents and learners). In Figure 1B, the box and whisker plots show the distribution of the number of questions correctly answered by all respondents and learners. In both plots, the horizontal red line within the box indicates the median, the “x” symbol represents the mean, the boxes indicate the IQR, and the vertical lines (whiskers) extend to the range of values, excluding outliers. The outliers are defined as values that fall outside a distance of 1.5 times the IQR from the upper and lower quartiles and are represented by empty circles. The respondents and learners were the HCPs who completed the pre- and postactivity questionnaires, respectively. For both activities, there was a statistically significant increase in the number of correctly answered questions from pre- to postactivity for all participants (touchMDT: median 4.0, IQR 3.0-5.0 to median 5.5, IQR 4.0-7.0; mean 4.00, SD 1.39 to mean 5.30, SD 1.56; *P*<.001 and touchPANEL DISCUSSION: median 4.0, IQR 4.0-5.0 to median 6.0, IQR 5.0-7.0; mean 4.32, SD 1.30 to mean 5.88, SD 1.49; *P*<.001; Figure 1). In subgroup analyses, significant increases in the number of correctly answered questions were observed irrespective of specialty or country for both activities and irrespective of years of experience for the touchPANEL DISCUSSION (Multimedia Appendices 12 and 13). Refer to Multimedia Appendix 14 for the responses to individual topics for the levels 3 to 4 questionnaires.

#### Level 5 (Performance)

A total of 50 respondents and 50 learners completed the level 5 questionnaire and the patient records questionnaire (Table 1). The dropout rate was 16% (8/50); therefore, 8 of the 50 learners were de novo HCPs who were matched to the dropouts.

##### Self-Reported

**Figure 2:**
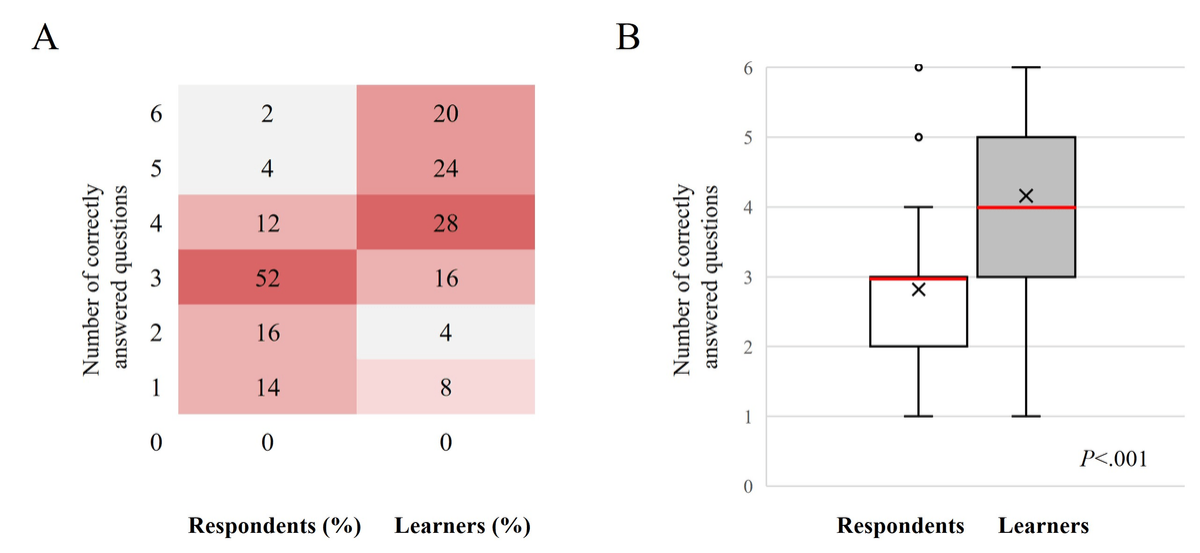
Summary of the number of correct responses for the level 5 outcomes questionnaire before and after the launch of touchMDT and touchPANEL DISCUSSION. (A) Heat maps show the proportion of respondents (n=50) and learners (n=50) who answered a specific number of questions correctly, as displayed by colors ranging from white (the lowest proportion of respondents and learners) to dark red (the highest proportion of respondents and learners). (B) Box and whisker plots show the distribution of the number of questions correctly answered by all respondents and learners. The horizontal red line within the box indicates the median, the “x” symbol represents the mean, the boxes indicate the IQR, and the vertical lines (whiskers) extend to the range of values, excluding outliers.

Preactivity, in total, 6% (3/50) of respondents answered at least 5 (83%) out of 6 questions with the best clinical option. This increased to 44% (22/50) of the learners after the activity (Figure 2). In Figure 2A, the heat maps show the proportion of respondents (n=50) and learners (n=50) who answered a specific number of questions correctly, as displayed by colors ranging from white (the lowest proportion of respondents and learners) to dark red (the highest proportion of respondents and learners). In Figure 2B, the box and whisker plots show the distribution of the number of questions correctly answered by all respondents and learners. The horizontal red line within the box indicates the median, the “x” symbol represents the mean, the boxes indicate the IQR, and the vertical lines (whiskers) extend to the range of values, excluding outliers. The outliers are defined as values that fall outside a distance of 1.5 times the IQR from the upper and lower quartiles and are represented by empty circles. The respondents and learners were HCPs who completed the pre- and postactivity questionnaires, respectively. There was a statistically significant increase in the number of correctly answered questions from pre- to postactivity for all participants (median 3.0, IQR 2.0-3.0 to median 4.0, IQR 3.0-5.0; mean 2.82, SD 1.08 to mean 4.16, SD 1.45; *P*<.001). Refer to Multimedia Appendix 15 for the responses to individual topics for the level 5 questionnaires.

In subgroup analyses of data by specialty, years of experience and country (Multimedia Appendix 16), increases in the number of questions with the correct clinical response were observed irrespective of specialty or years of experience, and in the subgroups of participants from Germany, Spain, and Italy.

##### Patient Record Data

The patient characteristics reported by the respondents and learners were broadly similar (Multimedia Appendix 17). The respondents and learners used a wide range of measures to determine the patient risk of recurrence (30 and 26 different combinations of measures, respectively; Multimedia Appendix 18). There was a significant difference between the number of respondents and learners selecting each measure (*χ*^2^_49_=508.474; *P*<.001) and the number of measures selected (*χ*^2^_56_=203.125; *P*<.001; Multimedia Appendix 19). The Ki-67 index use was >90% for both groups, and most of those who used the Ki-67 index reported patients who had tumors with a Ki-67 score of ≥20% (respondents: 30/48, 62.5% and learners: 30/47, 64%; Figure S1 in Multimedia Appendix 20). There was no significant difference between responders and learners regarding adjuvant therapy when analyzing patient record data by Ki-67 index, menopausal status, germline *BRCA* status, or the number of positive lymph nodes (Figure S2 in Multimedia Appendix 20).

### Self-Reported Confidence and Intention to Change Practice

Results from the levels 3 to 4 and level 5 questionnaires indicated modest increases in confidence in treating patients with EBC from pre- to postactivity, with the proportion of participants reporting that they felt moderately or extremely confident increasing from 72% (36/50) to 78% (39/50; *P*=.82) for touchMDT and 74% (37/50) to 86% (43/50; *P*=.49) for touchPANEL DISCUSSION and from 72% (36/50) to 80% (40/50; *P*=.90) for the level 5 questionnaire (Figure S3 in Multimedia Appendix 20).

At least half of all learners stated that they would make a change to their practice following their participation in the activities (levels 3 to 4 questionnaire: touchMDT—25/50, 50% and touchPANEL DISCUSSION—29/50, 58%; level 5 questionnaire: 26/50, 52%). The reasons given for not making a change were split between the need for more education (levels 3 to 4 questionnaire: touchMDT—12/50, 24% and touchPANEL DISCUSSION—7/50, 14%; level 5 questionnaire: 13/50, 26%) and practical limitations (levels 3 to 4 questionnaire: touchMDT—13/50, 26% and touchPANEL DISCUSSION—14/50, 28%; level 5 questionnaire: 11/50, 22%).

### Identification of Outstanding Educational Gaps

In ranking the unmet educational needs, touchMDT learners highlighted “strategies for implementing SDM in clinical practice” and “effective communication strategies in SDM to optimize patient outcomes” as key areas for future education, and touchPANEL DISCUSSION learners highlighted “using genetic and biomarker testing to guide therapy choice” and “individualization of treatment choice in high-risk hormone receptor–positive human epidermal growth factor receptor 2 negative (HR+ HER2–) EBC.” From the level 5 questionnaire, learners highlighted “using genetic and biomarker testing to guide therapy choice for patients with high-risk HR+ HER2- EBC” and “understanding the latest data on emerging treatments for high-risk HR+ HER2- EBC” as their most important unmet educational needs (Table S1 in Multimedia Appendix 20).

For the touchPANEL DISCUSSION, high levels of knowledge and competence were achieved for all 3 learning objectives. However, some unmet needs remained after participation in touchMDT, with 60% (30/50) and 36% (18/50) of the learners, respectively, being unable to demonstrate declarative knowledge of the current consensus on the use of patient decision aids to support SDM in breast cancer and being unable to demonstrate competence in supporting patients in understanding data and reaching a shared decision regarding neoadjuvant systemic therapy. In the level 5 questionnaire, ≥36% (18/50) of the learners were unable to provide the best clinical responses to the questions on reducing the patient burden (18/50, 36%), risk of recurrence (20/50, 40%), and chemotherapy treatment decisions (26/50, 52%).

## Discussion

### Principal Findings

This study evaluated the impact of 2 faculty-led, CME-accredited web-based learning activities on EBC. Participation in both activities resulted in high satisfaction scores and statistically significant improvements in self-reported knowledge, competence, and performance. Subgroup analyses indicated that the education was broadly beneficial, regardless of region, specialty, and experience. Patient record data analysis showed statistically significant differences in how the risk of recurrence was determined by responders and learners. The impact of the activities on therapeutic choice was limited, although this may be because of factors such as local guidelines, access to treatments, and individual patient characteristics. Approximately half of the learners (80/150, 53%) stated that they would change their practice in response to the educational material, further suggesting that the activities were valuable learning tools.

Participation in these web-based activities was considerably higher than could be achieved with traditional face-to-face programs and may also reflect the involvement of a multinational panel of recognized experts and the availability of translations. The latter is a major difference from traditional conference-based activities, as they are usually only available in 1 language, typically English.

Although many learners stated that they would change their practice, there remained a considerable number who were uncertain or who would not change their practice at the current time. The reasons for not changing practice were largely split between the requirement for more education and practical limitations. Although learners were not canvassed for specific details regarding practical limitations, we can speculate that they may relate to regional or national differences in institutional resources and structure or regulatory and reimbursement approvals of treatment. Indeed, documented barriers to change in clinical practice include institution-related issues such as flawed leadership and communication and insufficient organizational culture shift, and practical restrictions such as recently updated guidelines and access to emerging drugs [53]. In addition, a 2022 study on factors that influenced HCPs’ intent to put newly acquired learning into practice suggested that a lack of belief in one’s capabilities and the potential consequences of adopting new clinical behaviors can prevent HCPs from translating education into practice [54].

These data demonstrate the potential value of succinct web-based learning activities that are specifically designed to address knowledge gaps in complex and rapidly evolving medical fields. Although published data on outcomes following educational activities with similar methodologies remain limited, the results of this study are consistent with the results of a small number of previous studies in other disease areas [55-57]. Notably, the field of breast cancer is evolving rapidly, and it is essential for oncologists, particularly nonspecialists, to remain informed of the latest findings and recommendations [19-26]. Therefore, focused web-based learning activities may also be valuable for general oncologists to enable them to keep up-to-date with changes in specialist fields.

### Strengths and Limitations

There were several strengths associated with this study:

1. The activities provided a holistic curriculum and unbiased education focused on clearly defined learning objectives.
2. The activities were translated into several languages and were available on a free-to-access website, which likely increased their global accessibility.
3. A broad target audience was successfully reached, highlighting the importance of the multichannel publicity strategy and the inclusion of faculty from often underrepresented geographies (Latin America and Asia-Pacific).
4. The multidisciplinary team approach and inclusion of a survivor of breast cancer ensured that the activities were directly applicable to the participants’ daily practice; the emotive element can also aid learning and retention [58].
5. The format used in the touchPANEL DISCUSSION allowed the latest evidence to be put into context for practicing physicians.
6. The development of the education in a short time frame allowed for emerging data to be communicated to the HCPs potentially ahead of publications and conventional educational formats.
7. A major strength was using an objective measure of performance to complement the more subjective self-reported data.

This study also has several limitations:

1. The conclusions that could be drawn from the main and subgroup analyses were speculative and limited because of the relatively small sample size.
2. All medical educational studies are affected by inevitable self-selection bias; that is, the HCPs who feel they lack knowledge on a specific topic are more likely to participate and, therefore, show a bigger impact of the education than might be the case for a wider audience.
3. This study only evaluated outcomes following 2 specific CME activities in EBC, and the conclusions may not be generalizable to other web-based learning formats.
4. An independent samples method was used for the levels 3 to 4 questionnaires and the self-reported level 5 questionnaire to avoid pre-exposure bias; however, this meant that the direct impact of education on each learner could not be determined. Therefore, we cannot rule out the possibility that other sources of education may have contributed to the reported outcomes.

Despite these limitations, this study offers the first comprehensive assessment of such activities using an accepted methodology.

### Identification of Needs for Further Education in High-Risk EBC

Notably, only 30% (15/50) and 48% (24/50) of the touchMDT and touchPANEL DISCUSSION learners, respectively, answered all questions correctly in the levels 3 to 4 questionnaires. Similarly, only 20% (10/50) of the learners gave the best clinical response for all questions in the level 5 questionnaire. This may, in part, reflect a mixed audience but suggests that further education may be warranted to reinforce the content of these activities. To refine future educational activities, we identified several unmet needs during this study from the questions that were incorrectly answered after the activities. In addition, several self-reported educational gaps were identified, including implementing SDM in clinical practice, using genetic and biomarker testing to guide therapy choices, and understanding the latest data on emerging treatments.

### Future Directions

Limitations of traditional CME approaches in affecting changes in physician performance have been previously reported [59]; however, the optimal format for continuing educational activities in both breast cancer and the wider medical field remains to be fully defined. This study and other similar studies show evidence that more engaging approaches have the potential to improve the effectiveness of CME [55-57,60,61]. Evaluation of the long-term impacts of CME on HCP performance, alongside assessment against Moore’s Levels 6 and 7 [40] to determine the effect of education on patient (level 6) and community (level 7) health will add further value to CME programs in the future. Activity formats that may be particularly relevant include experts sharing insights drawn from experience and their own clinical practice, conversational and panel discussion formats, and expert interviews supplemented by vignettes of consultation strategies. Furthermore, web-based delivery may offer scope for developing more individualized approaches. Web-based activities can also be made available as enduring materials, providing a resource designed for optimal HCP accessibility. Future studies will need to be designed to evaluate the comparative effectiveness of these different approaches to CME. In addition, in future studies, a larger sample size will increase the statistical power of the analyses.

### Conclusions

Our study demonstrated that focused, free-to-access web-based CME activities on EBC were associated with improvements in HCP knowledge, competence, and self-reported performance. This study addressed the gaps in the provision of HCP-focused web-based IME programs that encompass recent advances in the high-risk EBC treatment landscape, the need for and approaches to effective SDM, and the gaps within the literature in terms of reporting outcomes from web-based CME activities. The identified unmet needs should be used to inform the design of future educational activities for this disease area. Further studies evaluating the long-term impact of education on HCP performance and on patient and community health will be valuable in defining the clinical impact of CME and the most effective channels for its delivery.

## Appendix

Multimedia Appendix 1 Activity development process.

Multimedia Appendix 2 Assessment of participation (level 1) and satisfaction (level 2).

Multimedia Appendix 3 Topics included in levels 3 and 4 and level 5 outcomes questionnaires.

Multimedia Appendix 4 Questions included in the levels 3 and 4 outcomes questionnaire for the touchMDT activity.

Multimedia Appendix 5 Questions included in the levels 3 and 4 outcomes questionnaire for the touchPANEL DISCUSSION activity.

Multimedia Appendix 6 Questions included in the level 5 outcomes questionnaire for the touchMDT and touchPANEL DISCUSSION activities.

Multimedia Appendix 7 Unmet educational need options included in levels 3 to 4 and level 5 questionnaires.

Multimedia Appendix 8 Single transferable vote system methodology.

Multimedia Appendix 9 Engagement results and demographics for the touchMDT and touchPANEL DISCUSSION activities.

Multimedia Appendix 10 Mean satisfaction scores of levels 2 to 4 questionnaire (out of a maximum of 5) completed by touchMDT and touchPANEL DISCUSSION learners.

Multimedia Appendix 11 Multichannel publicity reach and impact for the touchMDT and touchPANEL DISCUSSION activities.

Multimedia Appendix 12 Mean number of correct responses for the levels 3 and 4 outcomes questionnaire before and after the launch of touchMDT by (A) country, (B) years of experience, and (C) specialty subgroups of the respondents and learners.

Multimedia Appendix 13 Mean number of correct responses for the levels 3 and 4 outcomes questionnaire before and after the launch of touchPANEL DISCUSSION by (A) country, (B) level of experience, and (C) specialty of the respondents and learners.

Multimedia Appendix 14 Summary of correct responses for individual topics for the levels 3 and 4 outcomes questionnaire before and after the launch of (A) touchMDT and (B) touchPANEL DISCUSSION.

Multimedia Appendix 15 Summary of correct responses for individual topics for the level 5 outcomes questionnaire before and after the launch of touchMDT and touchPANEL DISCUSSION.

Multimedia Appendix 16 Mean number of correct responses for the level 5 outcomes questionnaire before and after the launch of touchMDT and touchPANEL DISCUSSION by (A) country, (B) level of experience, and (C) specialty of the respondents and learners.

Multimedia Appendix 17 Patient characteristics from patient records data (level 5) submitted by responders and learners for the touchPANEL DISCUSSION activity.

Multimedia Appendix 18 Combinations of measures used to determine patient risk for recurrence from patient records data (level 5) submitted by responders and learners for the touchPANEL DISCUSSION activity.

Multimedia Appendix 19 Measures used to determine patient risk for recurrence reported by respondents and learners in the level 5 patient records questionnaire.

Multimedia Appendix 20 Ki-67 use and Ki-67 index scores reported by respondents and learners in the level 5 patient records questionnaire (Figure S1); adjuvant or planned adjuvant therapy reported by respondents and learners in the level 5 patient records questionnaire (Figure S2); changes in self-reported confidence in (A) levels 3 to 4 questionnaires and (B) level 5 questionnaires following participation in the touchMDT and touchPANEL DISCUSSION activities (Figure S3); unmet educational needs identified by the touchMDT and touchPANEL DISCUSSION learners (Table S1).

## Abbreviations

Akt: protein kinase B
CME: continuing medical education
EBC: early breast cancer
HCP: health care professional
SDM: shared decision-making
touchIME: touch Independent Medical Education

## Data Availability

The data sets generated during and/or analyzed during this study are available from the corresponding author on reasonable request.
