# Supplementary material for: Assessing Knowledge, Competence, and Performance Following Web-Based Education on Early Breast Cancer Management: Health Care Professional Questionnaire Study and Anonymized Patient Records Analysis
Source: JMIR Form Res. 2024 Mar 21;8:e50931. doi: 10.2196/50931 (PMC10995792; doi:10.2196/50931)
Supplement: Multimedia Appendix 1 [file formative_v8i1e50931_app1.docx]

### Multimedia Appendix 1: Activity development process

An educational grant application was developed by medical directors at touchIME and submitted to Lilly, USA, LLC. The grant application included a review of educational gaps, a needs assessment, suggested expert faculty, learning objectives and agendas for the proposed educational activities. Target geographies were identified based on the educational gaps and needs. The grant application was based on a review of the relevant published literature and guidelines in high-risk early breast cancer (EBC) and feedback from expert international faculty. Lilly, USA, LLC, had no input into the faculty selection or content of the educational activities. Once the educational grant was awarded, faculty were invited to engage in the activity. Briefing calls were held with those who agreed to participate to discuss the activities and gain feedback on the educational gaps, learning objectives, proposed agenda and discussion points before moving forward. Once these details had been agreed, suggested slide content was drafted by touchIME based on data presented at the most recent relevant international conferences on breast cancer, including ESMO Breast Cancer 2022 and recent data from primary and secondary publications in peer-reviewed journals, as well as expert and patient opinions. Content was shared with expert faculty to review, verify, contribute to and amend. Faculty-approved content was used to record 10–15 minute videos that addressed the learning objectives. Both activities were recorded remotely using an online video-conferencing platform. Following video recording, touchIME drafted questionnaires to test the learners’ knowledge following the education. These were sent to faculty for review, verification, contribution and amendment. Activity content was quality checked throughout the development process by touchIME in-house editors and USF Health to ensure it was fair and balanced. Once finalized, all content was uploaded to touchONCOLOGY and launched as a free-to-access online educational activity.

The first activity was a touchMDT titled “How can shared decision-making be successfully integrated to optimize care of patients with high-risk early breast cancer?” The faculty comprised multidisciplinary specialists in breast cancer and shared decision making (SDM) (a medical oncologist, a specialist nurse and an oncology surgeon), plus a self-defined breast cancer survivor. The learning objectives were: (1) to recognize the importance and relevance of SDM in the context of EBC; (2) to evaluate the evidence for the effect that SDM has in optimizing outcomes for patients with breast cancer; and (3) to assess the challenges of implementing SDM and formulate strategies to overcome them.

The second activity was a touchPANEL DISCUSSION titled “New horizons in high-risk HR+ HER2- EBC: Risk stratification for early identification and novel treatment strategies”, with a faculty comprising leading breast cancer specialists. The learning objectives were: (1) to apply appropriate risk assessment and stratification strategies to predict patients’ likely risk of disease recurrence; (2) to evaluate the latest guidelines and treatment options for the management of high-risk HR+ HER2- EBC and assess the remaining unmet need; and (3) to interpret the evidence for novel therapeutic options for high-risk, HR+, HER2- EBC.
