# Supplementary material for: Assessing Knowledge, Competence, and Performance Following Web-Based Education on Early Breast Cancer Management: Health Care Professional Questionnaire Study and Anonymized Patient Records Analysis
Source: JMIR Form Res. 2024 Mar 21;8:e50931. doi: 10.2196/50931 (PMC10995792; doi:10.2196/50931)
Supplement: Multimedia Appendix 2 [file formative_v8i1e50931_app2.docx]

### Multimedia Appendix 2: Assessment of participation (Level 1) and satisfaction (Level 2)

Moore’s Level 1 (participation) was assessed separately for the two activities over the first 6 months after launch. Both the number of HCPs who engaged in the activity and the average time spent viewing the videos were measured by touchIME. Google Analytics captured geo-location, participant numbers and the overall average time HCPs had spent on the activity. Sub-analyses by country and specialty were also performed for the Level 1 variables. These data were collected from HCPs who viewed the activity via a log-in on their touchONCOLOGY.com account and from HCPs who completed the Level 3 and 4 outcomes questionnaires. The Level 2 questionnaire assessed satisfaction with the activity, and included the following six statements that were to be scored using a 1–5 Likert scale (where 5 is the highest satisfaction): this activity was of high quality; this activity met the stated educational objectives; the content was free from commercial bias; the presenters were knowledgeable and effective; the activity contained content relevant to my clinical practice; and the information presented is likely the help change my management strategies in this therapeutic area.
