# Supplementary material for: Assessing Knowledge, Competence, and Performance Following Web-Based Education on Early Breast Cancer Management: Health Care Professional Questionnaire Study and Anonymized Patient Records Analysis
Source: JMIR Form Res. 2024 Mar 21;8:e50931. doi: 10.2196/50931 (PMC10995792; doi:10.2196/50931)
Supplement: Multimedia Appendix 3 [file formative_v8i1e50931_app3.docx]

### Multimedia Appendix 3: Topics included in the Levels 3 and 4 and Level 5 outcomes questionnaires.

| **touchMDT** | **touchPANEL DISCUSSION** |
| --- | --- |
| **Levels 3 and 4** | |
| Implementing and supporting SDM for patients with high-risk EBC | Risk of disease recurrence in ER+ EBC |
| Management of psychological issues in breast cancer | Value of Ki-67 biomarker analysis |
| Role of partners/spouses, family and friends in patient decision making | Treatment options for patients with EBC and limited nodal burden |
| Use of PtDAs to support SDM in the use of neoadjuvant therapy in breast cancer | Benefits of targeted therapies for patients with high-risk EBC |
| **Level 5** | |
| Approaches for SDM in patients with high-risk EBC | |
| Management of anxiety and patient burden in high-risk EBC | |
| Risk of recurrence in EBC | |
| Treatment options for patients with high-risk EBC | |

**Abbreviation:** EBC, early breast cancer; ER+, estrogen receptor-positive; PtDA, patient decision aid; SDM, shared decision making; touchMDT, touch multidisciplinary team.
