# Supplementary material for: Assessing Knowledge, Competence, and Performance Following Web-Based Education on Early Breast Cancer Management: Health Care Professional Questionnaire Study and Anonymized Patient Records Analysis
Source: JMIR Form Res. 2024 Mar 21;8:e50931. doi: 10.2196/50931 (PMC10995792; doi:10.2196/50931)
Supplement: Multimedia Appendix 4 [file formative_v8i1e50931_app4.docx]

### Multimedia Appendix 4: Questions included in the Level 3 and 4 outcomes questionnaire for the touchMDT activity.

| **Questions/multiple choice answers*** |
| --- |
| 1. Your department is implementing SDM for your patients with high-risk early breast cancer. Which of the following actions would you adopt in your own practice to support decisional control for patients throughout the SDM process? *(Level 3)* 2. Avoid discussing cultural or religious issues surrounding SDM with your patients 3. Emphasize the importance of making decisions free from family influence 4. **Source appropriate decisional tools suited to the patient’s preferred learning style** 5. Double allocated consultation times to accommodate discussions |
| 1. Your 32-year-old patient with a 3.4 cm HR-positive tumor in her left breast expresses concerns surrounding how her planned mastectomy may change her body image and worries she may feel less feminine. What might you consider in the ongoing management of this patient? *(Level 4)* 2. Focus conversations on the practicalities of treatment to reduce additional stress 3. **Include a psychologist in the care team** 4. Advise the patient to discuss any non-treatment-related concerns with her family physician 5. Reduce the frequency of visits to minimize the patient burden |
| 1. Based on available research, when treating your patients with breast cancer, which of these is key for you to consider in terms of patients’ decisional control when using SDM approaches? *(Level 3)* 2. **Partners/spouses, family and friends may influence patient decision-making** 3. Educational level is the most important influencing factor in patient decision-making 4. Most patients entrust their physician with all the decisions 5. Most patients prefer complete autonomy in reaching their decisions |
| 1. Your patient with high-risk EBC is conflicted between the surgical options offered to her: breast-conserving surgery versus a mastectomy. Which of the following would you consider next to best support effective SDM? *(Level 4)*      1. Advise the patient to come to a decision with the help of family and friends 2. Advise the patient to consult online tools that support decision-making in breast cancer 3. **Assess the patient’s preferred learning style to identify an appropriate PtDA designed specifically to support surgical decisions** 4. Advise the patient that most of your patients with high-risk EBC opt for a mastectomy |
| 1. Which of the following statements best summarizes how PtDA use can influence outcomes amongst women navigating neoadjuvant therapy decisions in operable breast cancer? *(Level 3)* 2. It can reduce decisional regret, but not anxiety 3. **It can reduce decisional regret and anxiety** 4. It can improve quality of life, but has no effect on decisional outcomes 5. It can improve quality of life, but not anxiety |
| 1. Which of the following statements best reflects current consensus on the use of PtDAs to support SDM in breast cancer treatment and care? *(Level 3)*      1. PtDAs robustly validated by trial data are widely available for systemic treatment and surgical decisions 2. A well-designed PtDA is universally applicable to patients with shared disease characteristics 3. A well-designed PtDA is universally applicable to patients with shared cultural characteristics 4. **Better tools are needed to meaningfully assess the value of PtDAs** |
| 1. You are discussing treatment options with your patient with operable high-risk EBC. They express frustration in trying to understand the risks and benefits associated with NAST. Which of the following next steps would you consider most appropriate to support your patient in understanding the data and reaching a shared decision? *(Level 4)* 2. Explain the current guideline recommendations for NAST 3. Offer to schedule an appointment for her to meet the oncology nurse to discuss associated risks further 4. Share the latest research papers on NAST-associated survival outcomes 5. **Share and explain a diagram that illustrates risk data for progression or becoming inoperable while on NAST** |

*The correct answer is indicated in bold. Level 3 questions were structured to assess knowledge of guidelines and clinical trial data and how these may be applied in clinical practice, whereas Level 4 questions were structured as patient cases to directly assess competence in making the optimal clinical decision. Respondents and learners are defined as healthcare professionals who completed the pre- and post-activity questionnaires, respectively.

**Abbreviations:** EBC, early breast cancer; HR, hormone receptor; NAST, neoadjuvant systemic therapy; PtDA, patient decision aid; SDM, shared decision making.
