# Supplementary material for: Assessing Knowledge, Competence, and Performance Following Web-Based Education on Early Breast Cancer Management: Health Care Professional Questionnaire Study and Anonymized Patient Records Analysis
Source: JMIR Form Res. 2024 Mar 21;8:e50931. doi: 10.2196/50931 (PMC10995792; doi:10.2196/50931)
Supplement: Multimedia Appendix 5 [file formative_v8i1e50931_app5.docx]

### Multimedia Appendix 5: Questions included in the Level 3 and 4 outcomes questionnaire for the touchPANEL DISCUSSION activity.

| **Questions/multiple choice answers*** |
| --- |
| 1. Your patient with ER+ early breast cancer asks you about her risk of disease recurrence after finishing a 5-year course of endocrine therapy. Based on findings from a 2017 meta-analysis of ~63,000 women, how would you advise her? *(Level 3)* 2. Her risk of distant recurrence is dependent upon whether she underwent breast conserving surgery or mastectomy 3. Her risk of distant recurrence is dependent upon her age 4. **Her risk of distant recurrence is dependent upon factors including the original size of her tumor and number of nodes involved** 5. Her risk of distant recurrence is dependent upon her menopausal status |
| 1. According to the Ki-67 biomarker analysis from the monarchE study, which of the following statements is correct? *(Level 3)* 2. **Ki-67 was prognostic for disease recurrence but not predictive of therapeutic response** 3. Ki-67 was prognostic for disease recurrence and predictive of therapeutic response 4. Ki-67 was not prognostic for disease recurrence but was predictive of therapeutic response 5. Ki-67 was not prognostic for disease recurrence or predictive of therapeutic response |
| 1. Your 51-year-old patient is perimenopausal and has treatment-naïve, T2N1a, HR+ HER2- early breast cancer. She would like to optimize her cosmetic outcome after breast conserving surgery and is worried about lymphedema after axillary lymph node dissection. Ki-67 biomarker testing determines a risk score of 17%. What treatment approach would you recommend to this patient (assuming all options are available)? *(Level 4)* 2. Recommend proceeding directly to surgery followed by chemotherapy 3. **Recommend a genomic assay to determine whether neoadjuvant chemotherapy would be suitable** 4. Recommend a ctDNA-based liquid biopsy to determine whether neoadjuvant chemotherapy would be suitable 5. Recommend proceeding with neoadjuvant chemotherapy followed by surgery |
| 1. Your 66-year-old postmenopausal patient has high-risk HR+ HER2- early breast cancer with a germline BRCA1 mutation. She has had neoadjuvant chemotherapy, surgery and radiotherapy. Together you discuss initiating adjuvant endocrine therapy plus olaparib. Your patient asks if there is any benefit to adding olaparib. What will you advise based on the Olympia trial results in women with high-risk HER2- early breast cancer and a germline BRCA1/2 mutation? *(Level 4)* 2. **Olaparib has been shown to increase invasive and distant DFS vs placebo** 3. Olaparib has been shown to increase invasive DFS only vs placebo 4. Olaparib has been shown to increase distant DFS only vs placebo 5. Olaparib has not been shown to increase invasive or distant DFS vs placebo |
| 1. Your patient with HR+ HER2- early breast cancer has a Ki-67 score of 23%. She had previously received surgery and radiotherapy. You discuss initiating endocrine therapy with a CDK4/6 inhibitor, and your patient asks for information on palbociclib as her friend with metastatic breast cancer is currently ‘doing well’ on this medication. What will you tell her based on the PALLAS and PENELOPE-B trial result? *(Level 3)* 2. Palbociclib has been shown to improve DFS in women with HR+ HER2- early breast cancer and a Ki-67 ≤10% 3. Palbociclib has been shown to improve DFS in women with HR+ HER2- early breast cancer and a Ki-67 ≥10% 4. **Palbociclib has not yet been shown to improve DFS in women with HR+ HER2- early breast cancer** |
| 1. Your postmenopausal 59-year-old patient has T3N1a, grade 3, HR+ HER2- early breast cancer (no germline BRCA1/2 mutation). She has previously received neoadjuvant chemotherapy, breast conserving surgery and radiotherapy. She now requires endocrine therapy. Which therapy would you prescribe? *(Level 4)* 2. Tamoxifen plus ribociclib 3. Tamoxifen plus palbociclib 4. **An aromatase inhibitor plus abemaciclib** 5. An aromatase inhibitor plus palbociclib |
| 1. Oral SERDs are currently being investigated as a novel treatment option for HR+ HER2- early breast cancer. How do they work? *(Level 3)* 2. **They bind to the ER and induce ER degradation, preventing translocation into the nucleus and inhibiting transcription of ER-regulated genes** 3. They inhibit conversion of androgens into estrogens, decreasing estrogen binding to the ER 4. They bind to the ER, preventing transcription of ER-regulated genes following translocation into the nucleus 5. They degrade estrogen, preventing it from binding to the ER |

*The correct answer is indicated in bold. Level 3 questions were structured to assess respondents' and learners’ knowledge of guidelines and clinical trial data and how these may be applied in clinical practice, whereas Level 4 questions were structured as patient cases to directly assess the competence of respondents and learners in making the optimal clinical decision. Respondents and learners are defined as healthcare professionals who completed the pre- and post-activity questionnaires, respectively.

**Abbreviations:** CDK4/6, cyclin-dependent kinases 4 and 6; ctDNA, circulating tumor DNA; DFS, disease-free survival; ER, estrogen receptor; HER2, human epidermal growth factor receptor 2; HR, hormone receptor; SERD, selective estrogen receptor degrader; T2N1a, tumor diameter >2 to ≤5 cm; 1–3 positive lymph nodes; ≥1 area of spread >2 mm; T3N1, tumor diameter >5 cm; 1–3 positive lymph nodes; ≥1 area of spread >2 mm.
