# Supplementary material for: Assessing Knowledge, Competence, and Performance Following Web-Based Education on Early Breast Cancer Management: Health Care Professional Questionnaire Study and Anonymized Patient Records Analysis
Source: JMIR Form Res. 2024 Mar 21;8:e50931. doi: 10.2196/50931 (PMC10995792; doi:10.2196/50931)
Supplement: Multimedia Appendix 6 [file formative_v8i1e50931_app6.docx]

### Multimedia Appendix 6: Questions included in the Level 5 outcomes questionnaire for the touchMDT and touchPANEL DISCUSSION activities.

| **Questions/multiple choice answers*** |
| --- |
| 1. Your patient has just been diagnosed with high-risk HR-positive, HER2-negative early breast cancer. When discussing potential treatment options, which of the following approaches do you take to best support shared decision-making with your patient? 2. With all patients, use the latest clinical guidelines as a framework for discussions 3. With all patients, share the latest clinical trial evidence for the treatment options discussed 4. **Use an individualized approach, adapting your communication method to the patient’s preferred learning style and individual needs** |
| 1. Your patient has a large but operable HR-positive/HER2-negative breast tumor. During a consultation, they express anxiety around the effects that treatment may have on their body. Which of these next steps do you take to best support your patient in reaching a decision they are comfortable with? 2. Advise the patient that joining an online support group will help to ease her anxiety 3. Advise the patient that discussions with family and friends will help to ease her anxiety 4. Give the patient online resources with testimonials from others experiencing similar body image concerns 5. **Explore specific fears held by the patient and identify management strategies through shared decision-making** |
| 1. Your patient with early breast cancer expresses feeling overwhelmed by the number of different specialities involved in their care, and by having to repeatedly recount their medical history at each appointment. Which of the following approaches do you consider implementing to best reduce the patient burden while navigating their treatment journey? 2. Encourage the patient to keep a notebook recording information related to their treatment 3. Discuss the issue of informational burden for this patient at the next MDT meeting 4. **Refer the patient to a dedicated breast cancer nurse navigator or equivalent team member** 5. Encourage the patient to bring a family member or friend to each appointment |
| 1. Your postmenopausal 62-year-old patient was diagnosed with HR+ HER2- early breast cancer, with an original tumor size of 4 cm and 3 positive lymph nodes. She underwent chemotherapy and surgery and has just completed 5 years of endocrine therapy. She wants to know about her risk of recurrence in the future. How do you advise her (assuming all options are available)? 2. Based on tumor size and nodal status, her risk of distant recurrence is high 3. Based on tumor size and nodal status, her risk of distant recurrence is higher than someone with a smaller tumor and fewer positive nodes 4. **Her tumor size and nodal status indicate an intermediate risk, but a genomic or biomarker assay would help provide further information** |
| 1. Your premenopausal 43-year-old patient has T2N1a, HR+ HER2- early breast cancer. When discussing initial breast conserving surgery, she expresses concern about cosmetic outcomes and lymphoedema after axillary lymph node dissection. Ki-67 biomarker testing determines a risk score of 27%. What do you do (assuming all options are available)? 2. Proceed with surgery followed by adjuvant chemotherapy 3. **Proceed with a genomic assay to determine whether chemotherapy would be suitable choice for this patient** 4. Proceed with neoadjuvant chemotherapy followed by surgery |
| 1. Which of the following treatment options do you use most often in the adjuvant systemic setting for patients with high-risk, node-positive, HR+ HER2- early breast cancer and negative for germline BRCA1/2 mutations? 2. Chemotherapy followed by endocrine therapy (tamoxifen or an AI) 3. Endocrine therapy (tamoxifen or an AI) 4. **Endocrine therapy (tamoxifen or an AI) plus abemaciclib** |

*The best clinical option is indicated in bold.

**Abbreviations:** AI, aromatase inhibitor; HER2, human epidermal growth factor receptor-2; HR, hormone receptor; MDT, multidisciplinary team; T2N1a, tumor diameter >2 to ≤5 cm with 1–3 positive lymph nodes and ≥1 area of spread >2 mm.
