# Supplementary material for: Assessing Knowledge, Competence, and Performance Following Web-Based Education on Early Breast Cancer Management: Health Care Professional Questionnaire Study and Anonymized Patient Records Analysis
Source: JMIR Form Res. 2024 Mar 21;8:e50931. doi: 10.2196/50931 (PMC10995792; doi:10.2196/50931)
Supplement: Multimedia Appendix 7 [file formative_v8i1e50931_app7.docx]

### Multimedia Appendix 7: Unmet educational need options included in the Level 3–4 and Level 5 questionnaires.

| **touchMDT**  **(Level 3–4 questionnaire)** | **touchPANEL DISCUSSION**  **(Level 3–4 questionnaire)** | **Level 5 questionnaire** |
| --- | --- | --- |
| Strategies for implementing SDM in clinical practice | Using genetic and biomarker testing to guide therapy choice for patients with high-risk HR+ HER2- early breast cancer | Strategies for implementing SDM in clinical practice |
| Effective communication strategies in SDM to optimize patient outcomes | Applying the latest guideline recommendations for management of patients with high-risk HR+ HER2- early breast cancer | Selecting and integrating patient decision aids to support meaningful SDM |
| Selecting and integrating patient decision aids to support meaningful SDM | Individualization of treatment choice for patients with high-risk HR+ HER2- early breast cancer | Managing SDM in the digital era/optimizing telemedicine consultations to address SDM needs |
| Managing SDM in the digital era/optimizing telemedicine consultations to address SDM needs | Understanding the latest data on emerging treatments for high-risk HR+ HER2- early breast cancer | Using genetic and biomarker testing to guide therapy choice for patients with high-risk HR+ HER2- early breast cancer |
|  |  | Applying the latest guideline recommendations for management of patients with high-risk HR+ HER2- early breast cancer |
|  |  | Understanding the latest data on emerging treatments for high-risk HR+ HER2- early breast cancer |

**Abbreviations:** HER2-, human epidermal growth factor receptor 2 negative; HR+, hormone receptor positive; SDM, shared decision making.
