# Supplementary material for: Assessing Knowledge, Competence, and Performance Following Web-Based Education on Early Breast Cancer Management: Health Care Professional Questionnaire Study and Anonymized Patient Records Analysis
Source: JMIR Form Res. 2024 Mar 21;8:e50931. doi: 10.2196/50931 (PMC10995792; doi:10.2196/50931)
Supplement: Multimedia Appendix 8 [file formative_v8i1e50931_app8.docx]

### Multimedia Appendix 8: Single transferable vote system methodology.

In the first round of voting, all first-choice votes are counted to determine the highest ranked educational gap for the participants; in the second round, all second-choice votes are counted to determine the second highest ranked educational gap. Any first-choice votes not from the winning option in the first round are also counted in the second round. Rounds of voting continue until all options have been placed in order.
