# Supplementary material for: Assessing Knowledge, Competence, and Performance Following Web-Based Education on Early Breast Cancer Management: Health Care Professional Questionnaire Study and Anonymized Patient Records Analysis
Source: JMIR Form Res. 2024 Mar 21;8:e50931. doi: 10.2196/50931 (PMC10995792; doi:10.2196/50931)
Supplement: Multimedia Appendix 9 [file formative_v8i1e50931_app9.docx]

### Multimedia Appendix 9: Engagement results and demographics for the touchMDT and touchPANEL DISCUSSION activities.

|  | **touchMDT** | **touchPANEL DISCUSSION** |
| --- | --- | --- |
| **Participant engagement, *N*** | 7,047 | 8,989 |
| **Countries reached, *N*** | 24 | 25 |
| **Mean length of participation, minutes** | 09:07 | 06:11 |
| **Specialty, *n* (%)** |  |  |
| Oncologist | 5,567 (79) | 8,180 (91) |
| Radiologist | 987 (14) | 809 (9) |
| Oncology nurse | 352 (5) | N/A |
| Pathologist | 141 (2) | N/A |
| **Country^a^, *n* (%)** |  |  |
| Italy | 1,621 (23) | 845 (9) |
| Portugal | 1,198 (17) | 2,925 (33) |
| Brazil | 997 (14) | 3,222 (36) |
| Japan | 775 (11) | 239 (3) |
| France | 775 (11) | 103 (1) |
| Spain | 600 (9) | 251 (3) |
| United States | 242 (3) | 145 (2) |
| Switzerland | 125 (2) | 9 (<1) |
| Taiwan | N/A | 693 (8) |

Data were collected on 29 September 2022 and 21 November 2022, 6 months after launch of the touchMDT and touchPANEL DISCUSSION activities, respectively. ^a^Country where the participant was based at the time of completing the activity. Data are reported for countries represented by ≥2% of participants for at least one activity.

**Abbreviations:** N/A, not applicable; touchMDT, touch multidisciplinary team..
