# Supplementary material for: Assessing Knowledge, Competence, and Performance Following Web-Based Education on Early Breast Cancer Management: Health Care Professional Questionnaire Study and Anonymized Patient Records Analysis
Source: JMIR Form Res. 2024 Mar 21;8:e50931. doi: 10.2196/50931 (PMC10995792; doi:10.2196/50931)
Supplement: Multimedia Appendix 10 [file formative_v8i1e50931_app10.docx]

### Multimedia Appendix 10: Level 2–4 questionnaire mean satisfaction scores (out of a maximum of 5) from touchMDT and touchPANEL DISCUSSION learners.

| **Satisfaction outcomes** | **touchMDT** | **touchPANEL DISCUSSION** |
| --- | --- | --- |
| **High quality of the activity** | 4.1 | 4.3 |
| **Knowledgeable and  effective presenters** | 4.3 | 4.4 |
| **Met the stated  learning objectives** | 4.1 | 4.5 |
| **Relevant to clinical practice** | 4.1 | 4.5 |
| **Will impact on management strategies** | 4.0 | 4.2 |

Data were collected on 29 September 2022 and 21 November 2022, 6 months after launch of the touchMDT and touchPANEL DISCUSSION activities, respectively. Learners are defined as healthcare professionals who completed the post-activity questionnaires.

**Abbreviation:** touchMDT, touch multidisciplinary team.
