# Supplementary material for: Assessing Knowledge, Competence, and Performance Following Web-Based Education on Early Breast Cancer Management: Health Care Professional Questionnaire Study and Anonymized Patient Records Analysis
Source: JMIR Form Res. 2024 Mar 21;8:e50931. doi: 10.2196/50931 (PMC10995792; doi:10.2196/50931)
Supplement: Multimedia Appendix 11 [file formative_v8i1e50931_app11.docx]

### Multimedia Appendix 11: Multichannel publicity reach and impact for the touchMDT and touchPANEL DISCUSSION activities.

| **Channels** | **touchMDT** | **touchPANEL DISCUSSION** |
| --- | --- | --- |
| **Facebook** |  |  |
| Reach^a^ | 636,191 | 982,270 |
| Impressions^b^ | 2,430,247 | 1,918,036 |
| **Twitter** |  |  |
| Impressions^b^ | 569,293 | 569,293 |
| **LinkedIn** |  |  |
| Impressions^b^ | 108,129 | 69,271 |
| **touchONCOLOGY** |  |  |
| Impressions^b^ | 32,574 | 10,981 |
| **Emails** |  |  |
| Delivered | 3,126 | 16,858 |
| Opened | 747 | 4,489 |

^a^”Reach” is defined as the number of people who had any content from the campaign or about the campaign enter their screen; ^b^”Impressions” is defined as the number of times any content from the campaign or about the campaign entered a person's screen. Data were collected on 29 September 2022 and 21 November 2022, 6 months after launch of the touchMDT and touchPANEL DISCUSSION
