# Supplementary material for: Assessing Knowledge, Competence, and Performance Following Web-Based Education on Early Breast Cancer Management: Health Care Professional Questionnaire Study and Anonymized Patient Records Analysis
Source: JMIR Form Res. 2024 Mar 21;8:e50931. doi: 10.2196/50931 (PMC10995792; doi:10.2196/50931)
Supplement: Multimedia Appendix 12 [file formative_v8i1e50931_app12.docx]

### Multimedia Appendix 12: Mean number of correct responses for the Level 3 and 4 outcomes questionnaire before and after the launch of touchMDT by (A) country, (B) years of experience and (C) specialty subgroups of the respondents and learners.

Respondents and learners are defined as healthcare professionals who completed the pre- and post-activity questionnaires, respectively. The *P*-values indicate the difference between baseline and follow-up values across groups.

**Abbreviation:** touchMDT, touch multidisciplinary team.

**(A)**

**
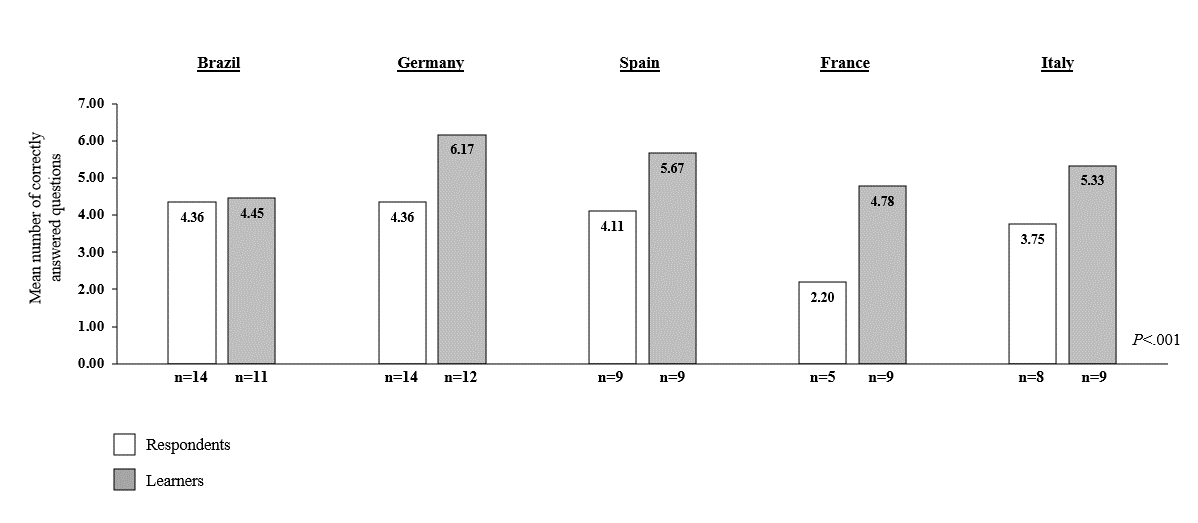
**

**(B)**

**
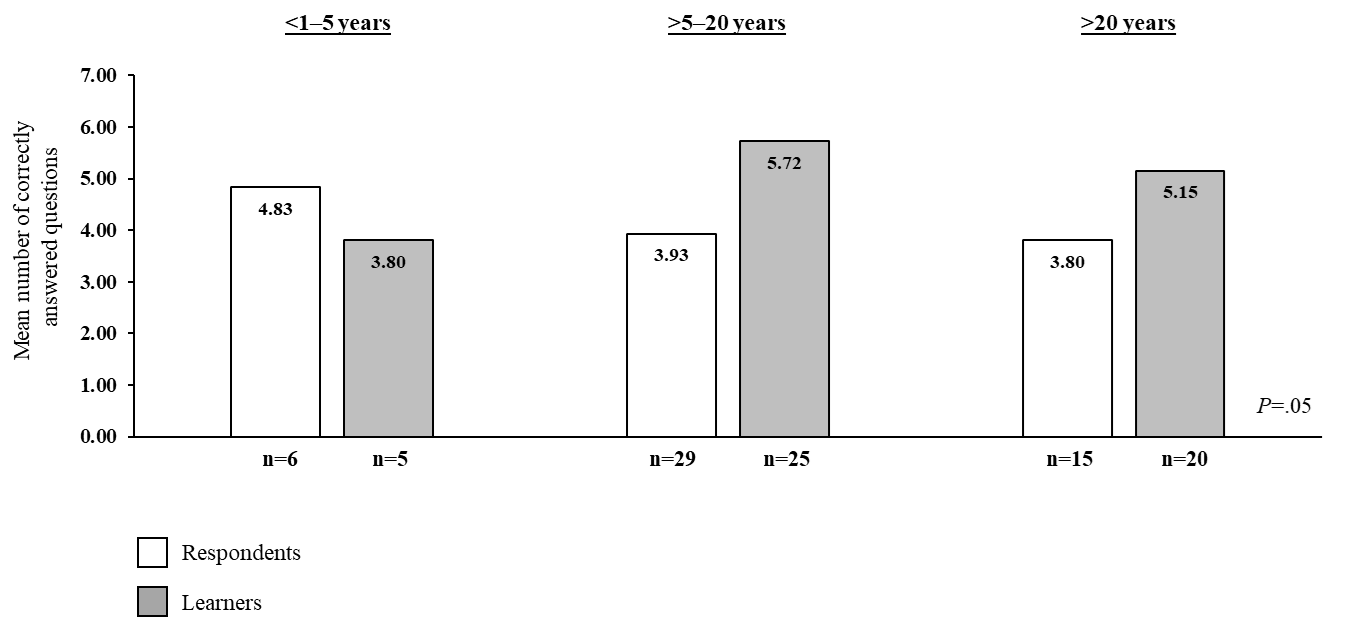
**

**(C)**

**
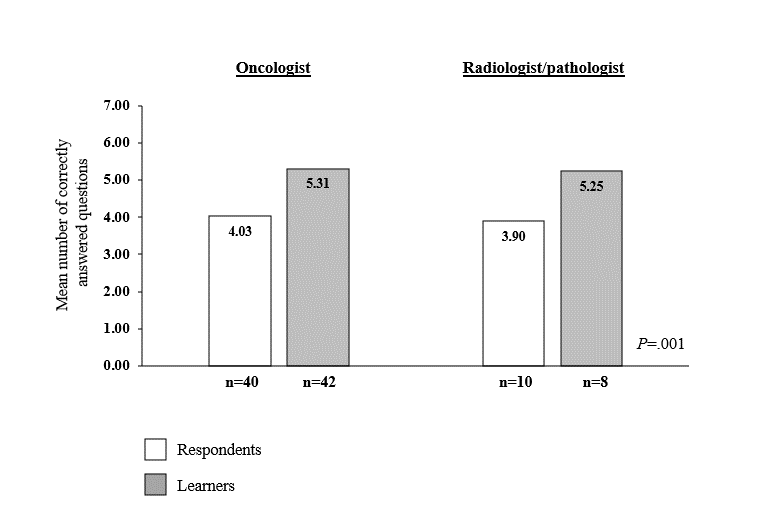
**
