# Supplementary material for: Assessing Knowledge, Competence, and Performance Following Web-Based Education on Early Breast Cancer Management: Health Care Professional Questionnaire Study and Anonymized Patient Records Analysis
Source: JMIR Form Res. 2024 Mar 21;8:e50931. doi: 10.2196/50931 (PMC10995792; doi:10.2196/50931)

### Multimedia Appendix 13: Mean number of correct responses for the Level 3 and 4 outcomes questionnaire before and after the launch of touchPANEL DISCUSSION by (A) country, (B) level of experience and (C) specialty of the respondents and learners.

Respondents and learners are defined as healthcare professionals who completed the pre- and post-activity questionnaires, respectively. The *P*-values indicate the difference between responders and learners across subgroups.

**Abbreviation:** touchMDT, touch multidisciplinary team.

**(A)**


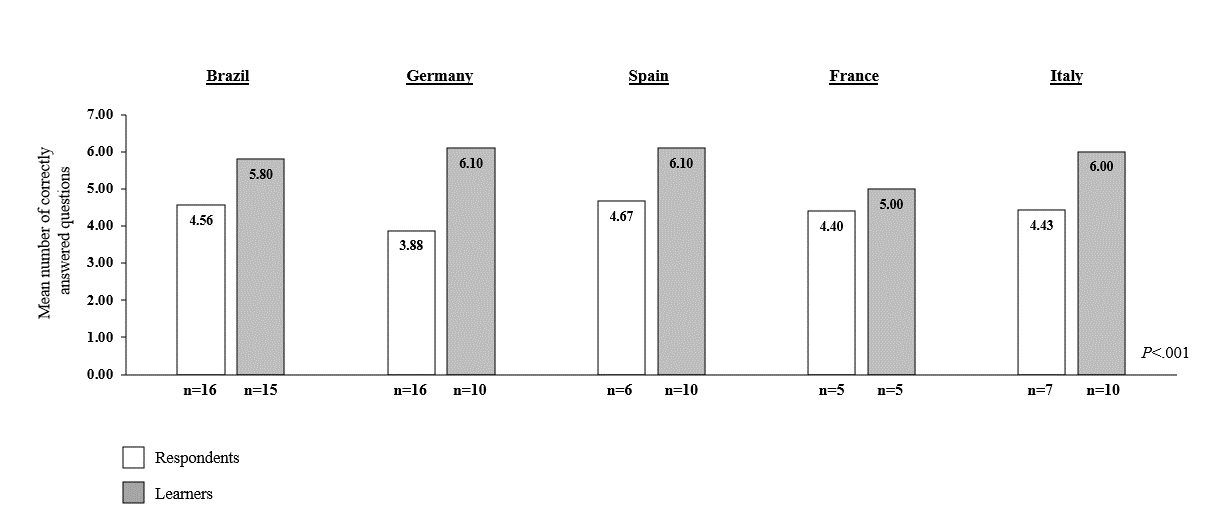


**(B)**


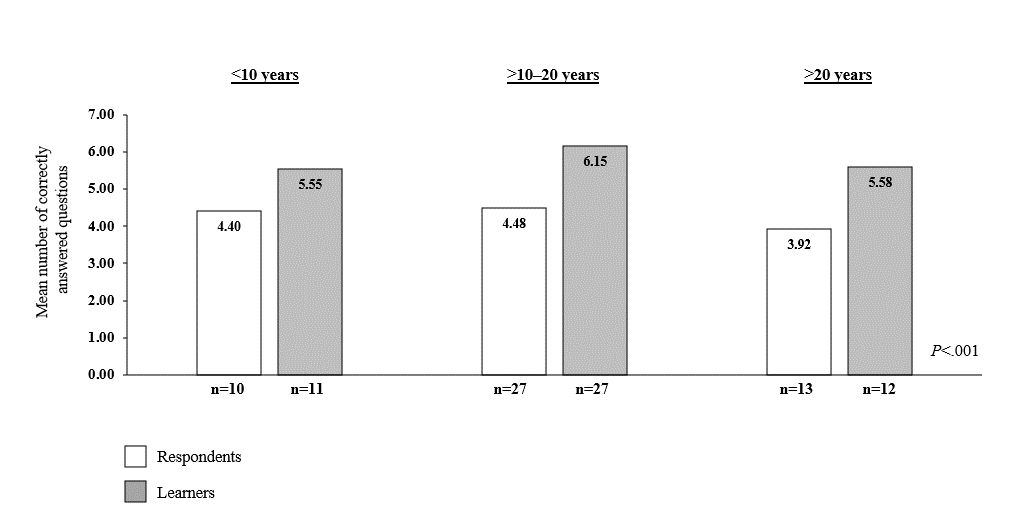


**(C)**


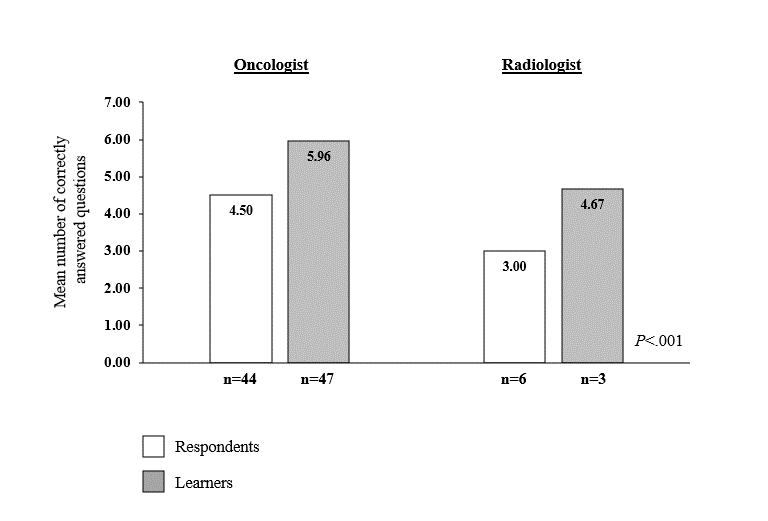

Supplement: Multimedia Appendix 13 [file formative_v8i1e50931_app13.docx]
