# Supplementary material for: Assessing Knowledge, Competence, and Performance Following Web-Based Education on Early Breast Cancer Management: Health Care Professional Questionnaire Study and Anonymized Patient Records Analysis
Source: JMIR Form Res. 2024 Mar 21;8:e50931. doi: 10.2196/50931 (PMC10995792; doi:10.2196/50931)

### Multimedia Appendix 14: Summary of correct responses for individual topics for the Level 3 and 4 outcomes questionnaire before and after the launch of (A) touchMDT and (B) touchPANEL DISCUSSION.

Bar graphs show the percentage of respondents (*N*=50) and learners (*N*=50) who answered each question correctly. Numbers within bars indicate their value. Respondents and learners are defined as healthcare professionals who completed the pre- and post-activity questionnaires, respectively.

**Abbreviations:** NAST, neoadjuvant systemic therapy; PARP, poly (ADP-ribose) polymerase; PtDA, patient decision aid; SDM, shared decision making; SERD, selective estrogen receptor degrader; touchMDT, touch multidisciplinary team.

**(A)**

**
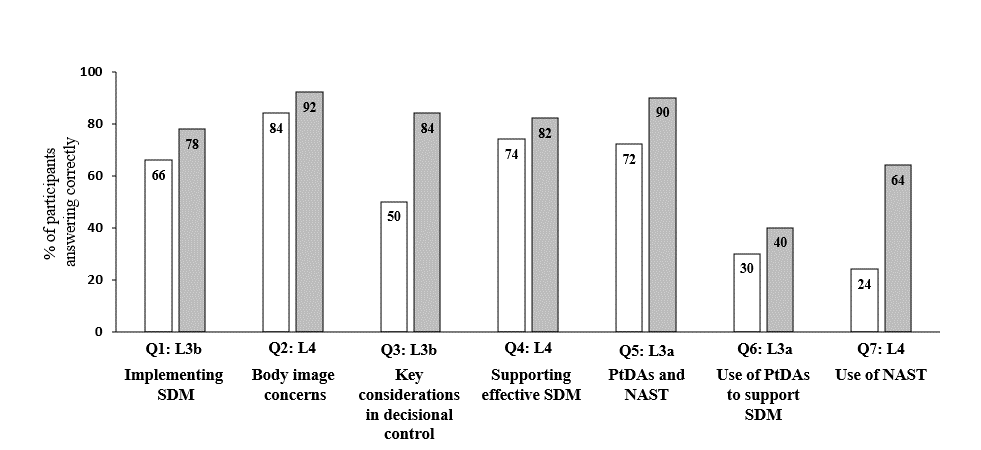
**

**(B)**


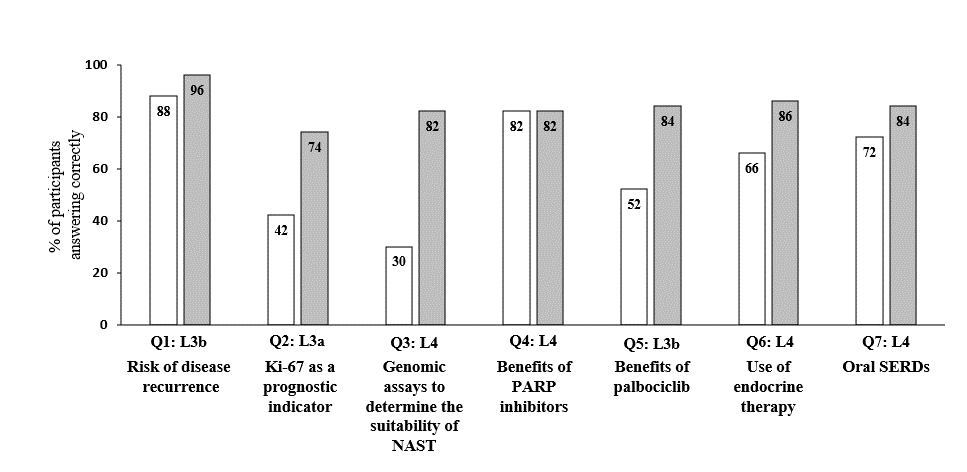

Supplement: Multimedia Appendix 14 [file formative_v8i1e50931_app14.docx]
