# Supplementary material for: Assessing Knowledge, Competence, and Performance Following Web-Based Education on Early Breast Cancer Management: Health Care Professional Questionnaire Study and Anonymized Patient Records Analysis
Source: JMIR Form Res. 2024 Mar 21;8:e50931. doi: 10.2196/50931 (PMC10995792; doi:10.2196/50931)
Supplement: Multimedia Appendix 15 [file formative_v8i1e50931_app15.docx]

### Multimedia Appendix 15: Summary of correct responses for individual topics for the Level 5 outcomes questionnaire before and after the launch of touchMDT and touchPANEL DISCUSSION.

Bar graphs show the percentage of respondents (*N*=50) and learners (*N*=50) who answered each question correctly. Numbers within bars indicate their value. Respondents and learners are defined as healthcare professionals who completed the pre- and post-activity questionnaires, respectively.

**
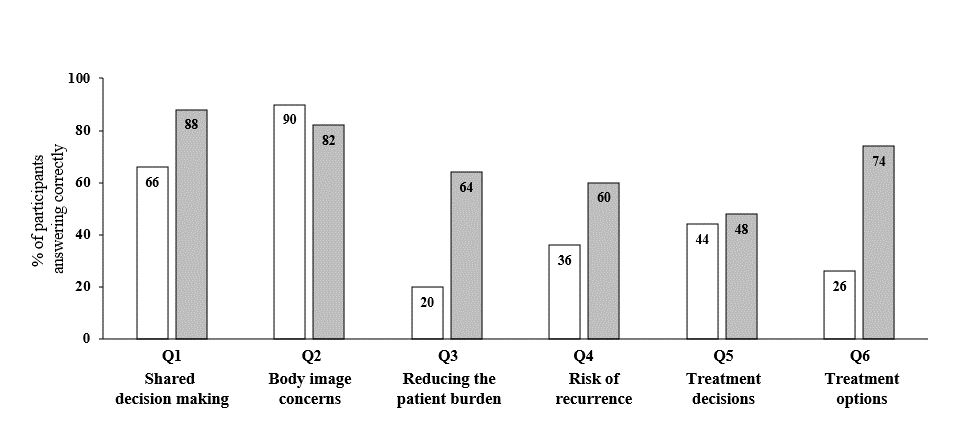
**
