# Supplementary material for: Assessing Knowledge, Competence, and Performance Following Web-Based Education on Early Breast Cancer Management: Health Care Professional Questionnaire Study and Anonymized Patient Records Analysis
Source: JMIR Form Res. 2024 Mar 21;8:e50931. doi: 10.2196/50931 (PMC10995792; doi:10.2196/50931)
Supplement: Multimedia Appendix 17 [file formative_v8i1e50931_app17.docx]

### Multimedia Appendix 17: Patient characteristics from patient records data (Level 5) submitted by responders and learners for the touchPANEL DISCUSSION activity.

| Patient characteristics | Responders  (*N*=50) | Learners  (*N*=50) |
| --- | --- | --- |
| Age, years | 52.86 | 54.14 |
| Date of diagnosis, *n* (%) |  |  |
| Pre-2020 | 1 (2) | 2 (4) |
| January–June 2020 | 3 (6) | 1 (2) |
| July–December 2020 | 0 | 0 |
| January–June 2021 | 4 (8) | 0 |
| July–December 2021 | 4 (8) | 5 (10) |
| January–June 2022 | 38 (76) | 11 (22) |
| July–December 2022 | 0 | 31 (62) |
| BRCA mutation status, *n* (%) |  |  |
| Negative | 39 (78) | 45 (90) |
| Positive | 11 (22) | 5 (10) |
| Menopausal status, *n* (%) |  |  |
| Premenopausal | 24 (48) | 15 (30) |
| Postmenopausal | 26 (52) | 35 (70) |
| Positive axillary lymph nodes, *n* (%) |  |  |
| 0 | 10 (20) | 8 (16) |
| 1–3 | 32 (64) | 36 (72) |
| ≥4 | 8 (16) | 6 (12) |
| Histopathological grade at diagnosis, *n* (%) |  |  |
| 1 | 1 (2) | 3 (6) |
| 2 | 36 (72) | 31 (62) |
| 3 | 13 (26) | 14 (28) |
| Grade cannot be assessed | 0 | 2 (4) |
| Pathologic tumor size, *n* (%) |  |  |
| ≤2 cm | 19 (38) | 17 (34) |
| >2 to ≤5 cm | 29 (58) | 32 (64) |
| >5 cm | 2 (4) | 1 (2) |
| Ki-67 index assessed, n (%) | 48 (96) | 47 (94) |
| <20% | 18 (37.5) | 17 (36) |
| ≥20% | 30 (62.5) | 30 (64) |
| TNM stage (derived), *n* (%) |  |  |
| IA | 7 (14) | 4 (8) |
| IIA | 11 (22) | 12 (24) |
| IIB | 16 (32) | 19 (38) |
| IIIA | 10 (20) | 10 (20) |
| IIIB | 4 (8) | 2 (4) |
| IIIC | 2 (4) | 3 (6) |
| Prior chemotherapy, *n* (%) |  |  |
| None | 33 (66) | 31 (62) |
| Neoadjuvant chemotherapy | 9 (18) | 15 (30) |
| Adjuvant chemotherapy | 8 (16) | 4 (8) |
| Frontline therapy, *n* (%) |  |  |
| Neoadjuvant chemotherapy | 21 (42) | 26 (52) |
| Neoadjuvant endocrine therapy | 3 (6) | 4 (8) |
| Surgery | 26 (52) | 20 (40) |
| Surgery and radiotherapy, *n* (%) |  |  |
| Yes | 29 (58) | 36 (72) |
| No | 2 (4) | 4 (8) |
| No, but planned | 19 (38) | 10 (20) |
| Most recent visit, *n* (%) |  |  |
| February–April | 8 (16) | 0 |
| May–July | 42 (84) | 0 |
| August–October | 0 | 14 (28) |
| November | 0 | 36 (72) |

Data were collected on 18 November 2022, 6 months after launch of the touchPANEL DISCUSSION activity. Respondents and learners are defined as healthcare professionals who completed the pre- and post-activity questionnaires, respectively.

**Abbreviation:** TNM, tumor, node, metastasis.
