# Supplementary material for: Assessing Knowledge, Competence, and Performance Following Web-Based Education on Early Breast Cancer Management: Health Care Professional Questionnaire Study and Anonymized Patient Records Analysis
Source: JMIR Form Res. 2024 Mar 21;8:e50931. doi: 10.2196/50931 (PMC10995792; doi:10.2196/50931)
Supplement: Multimedia Appendix 18 [file formative_v8i1e50931_app18.docx]

### Multimedia Appendix 18: Combinations of measures used to determine patient risk for recurrence from patient records data (Level 5) submitted by responders and learners for the touchPANEL DISCUSSION activity.

| **Combinations of measures used to determine patient risk for recurrence, n** | **Respondents (*N*=50)** | **Learners  (*N*=50)** |
| --- | --- | --- |
| TNM stage, immunohistochemistry (tumor marker status), Ki-67 index, original tumor size and number of nodes involved | 3 | 1 |
| Ki-67 index | 3 | 2 |
| TNM stage, histological grade, Ki-67 index, original tumor size and number of nodes involved | 3 | 3 |
| TNM stage | 3 | 5 |
| Age, menopausal status, TNM stage, histological grade, Ki-67 index | 2 | 0 |
| Age, menopausal status, TNM stage, histological grade, immunohistochemistry (tumor marker status), genomic assays, Ki-67 index, original tumor size and number of nodes | 2 | 4 |
| Original tumor size and number of nodes involved | 2 | 7 |
| TNM stage, Ki-67 index, original tumor size and number of nodes involved | 2 | 1 |
| TNM stage, genomic assays, Ki-67 index | 2 | 1 |
| Age, TNM stage, histological grade, immunohistochemistry (tumor marker status), genomic assays | 1 | 0 |
| Genomic assays | 1 | 0 |
| TNM stage, immunohistochemistry (tumor marker status) | 1 | 0 |
| TNM stage, histological grade, Ki-67 index | 1 | 1 |
| Age, TNM stage, histological grade, Ki-67 index | 1 | 0 |
| Age, TNM stage, histological grade, immunohistochemistry (tumor marker status), original tumor size and number of nodes involved | 1 | 0 |
| Age, menopausal status, TNM stage, histological grade, Ki-67 index, original tumor size and number of nodes involved | 1 | 0 |
| Age, menopausal status, TNM stage, histological grade, immunohistochemistry (tumor marker status), Ki-67 index, original tumor size and number of nodes involved | 1 | 0 |
| TNM stage, histological grade, original tumor size and number of nodes involved | 1 | 1 |
| Age, menopausal status, TNM stage, Ki-67 index, original tumor size and number of nodes involved | 1 | 0 |
| Age | 1 | 0 |
| TNM stage, histological grade, immunohistochemistry (tumor marker status), Ki-67 index, original tumor size and number of nodes involved | 1 | 2 |
| Age, menopausal status, TNM stage, histological grade, immunohistochemistry (tumor marker status), Ki-67 index | 1 | 1 |
| Menopausal status, TNM stage, histological grade, Ki-67 index, original tumor size and number of nodes involved | 1 | 0 |
| Histological grade, Ki-67 index | 1 | 0 |
| TNM stage, histological grade, immunohistochemistry (tumor marker status), Ki-67 index | 1 | 1 |
| Histological grade, Ki-67 index, original tumor size and number of nodes involved | 1 | 0 |
| TNM stage, original tumor size and number of nodes involved | 1 | 2 |
| Age, menopausal status, genomic assays | 1 | 0 |
| TNM stage, histological grade, immunohistochemistry (tumor marker status), genomic assays, Ki-67 index, original tumor size and number of nodes involved | 1 | 0 |
| Age, TNM stage, histological grade, immunohistochemistry (tumor marker status), Ki-67 index | 1 | 0 |
| Genomic assays, Ki-67 index, original tumor size and number of nodes involved | 0 | 1 |
| Age, menopausal status, TNM stage, histological grade, immunohistochemistry (tumor marker status), original tumor size and number of nodes involved | 0 | 1 |
| Menopausal status, TNM stage, immunohistochemistry (tumor marker status), Ki-67 index | 0 | 1 |
| Ki-67 index, original tumor size and number of nodes involved | 0 | 1 |
| TNM stage, histological grade, immunohistochemistry (tumor marker status), genomic assays, Ki-67 index | 0 | 1 |
| Menopausal status, TNM stage, histological grade, genomic assays, based on Ki-67 index | 0 | 1 |
| Menopausal status | 0 | 1 |
| Age, TNM stage, Ki-67 index | 0 | 1 |
| TNM stage, immunohistochemistry (tumor marker status), genomic assays | 0 | 1 |
| TNM stage, histological grade, immunohistochemistry (tumor marker status) | 0 | 1 |
| Immunohistochemistry (tumor marker status) | 0 | 1 |
| Immunohistochemistry (tumor marker status), genomic assays, Ki-67 index | 0 | 1 |
| No | 7^a^ | 6^b^ |

^a^Reasons were as follows: “High risk evident”, “No reason”, “It has not been done yet but it will be done, since it was not going to modify the treatment planned at the beginning”, “Rejection of the patient”, “No routine”, “No consequence” and “Wasn't the topic today” (*n*=1 for each response); ^b^Reasons were as follows: “Intermediate is assumed”, “No routine”, “No wish”, “Not made by default”, “Not routinely determined” and “Not yet” (*n*=1 for each response).

Data were collected on 18 November 2022, 6 months after launch of the touchPANEL DISCUSSION activity. Respondents and learners are defined as healthcare professionals who completed the pre- and post-activity questionnaires, respectively.

**Abbreviation:** TNM, tumor, node, metastasis.
