# Supplementary material for: Assessing Knowledge, Competence, and Performance Following Web-Based Education on Early Breast Cancer Management: Health Care Professional Questionnaire Study and Anonymized Patient Records Analysis
Source: JMIR Form Res. 2024 Mar 21;8:e50931. doi: 10.2196/50931 (PMC10995792; doi:10.2196/50931)
Supplement: Multimedia Appendix 19 [file formative_v8i1e50931_app19.docx]

### Multimedia Appendix 19: Measures used to determine patient risk for recurrence reported by respondents and learners in the Level 5 patient records questionnaire.

Bar graphs show the percentage of respondents (*N*=50) and learners (*N*=50) who selected (A) each individual measure and (B) either 0, 1–4 or 5–8 measures to determine patient risk for recurrence. Numbers within bars indicate their value. Respondents and learners are defined as healthcare professionals who completed the pre- and post-activity questionnaires, respectively.

**(A)**

**
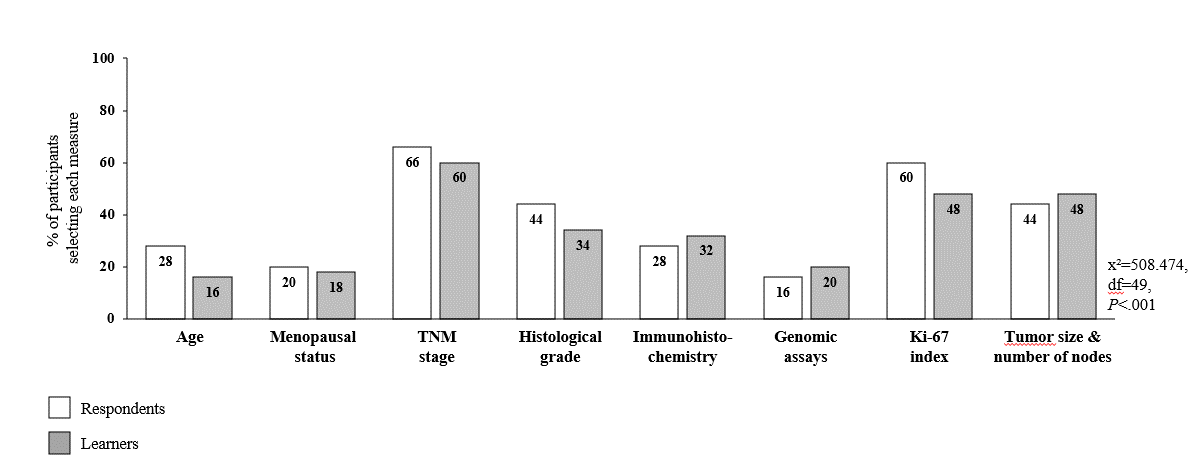
**

**(B)**

**
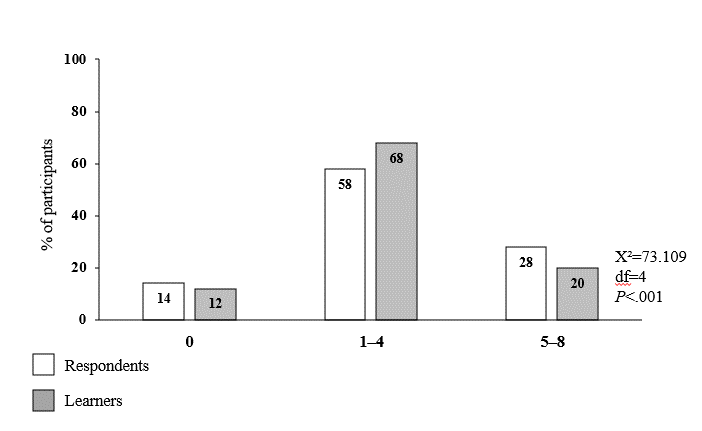
**
