# Supplementary material for: Assessing Knowledge, Competence, and Performance Following Web-Based Education on Early Breast Cancer Management: Health Care Professional Questionnaire Study and Anonymized Patient Records Analysis
Source: JMIR Form Res. 2024 Mar 21;8:e50931. doi: 10.2196/50931 (PMC10995792; doi:10.2196/50931)
Supplement: Multimedia Appendix 20 [file formative_v8i1e50931_app20.docx]

### Multimedia Appendix 20

### Figure S1. Ki-67 use and Ki-67 index scores reported by respondents and learners in the Level 5 patient records questionnaire.

Bar graphs show the percentage of respondents (*N*=50) and learners (*N*=50) who reported (A) Ki-67 use and (B) Ki-67 index scores reported by those who used Ki-67 index. Numbers within bars indicate their value. Respondents and learners are defined as healthcare professionals who completed the pre- and post-activity questionnaires, respectively.

**(A)**

**
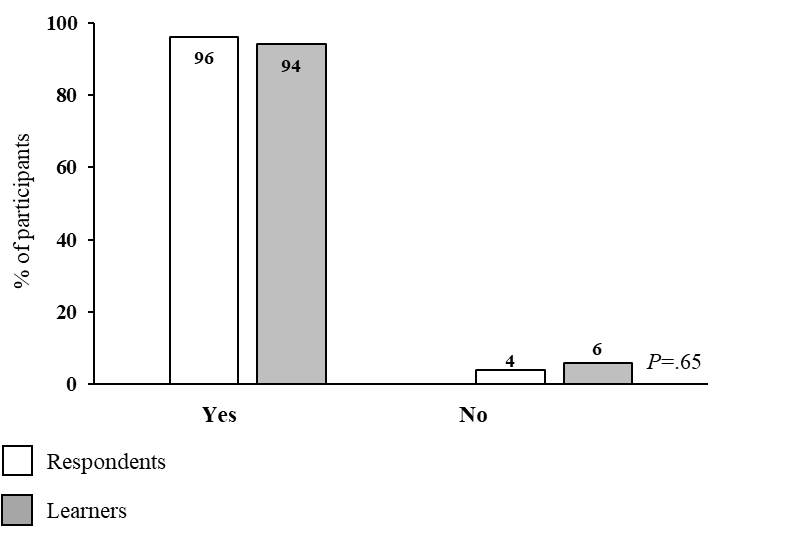
**

**(B)**

**
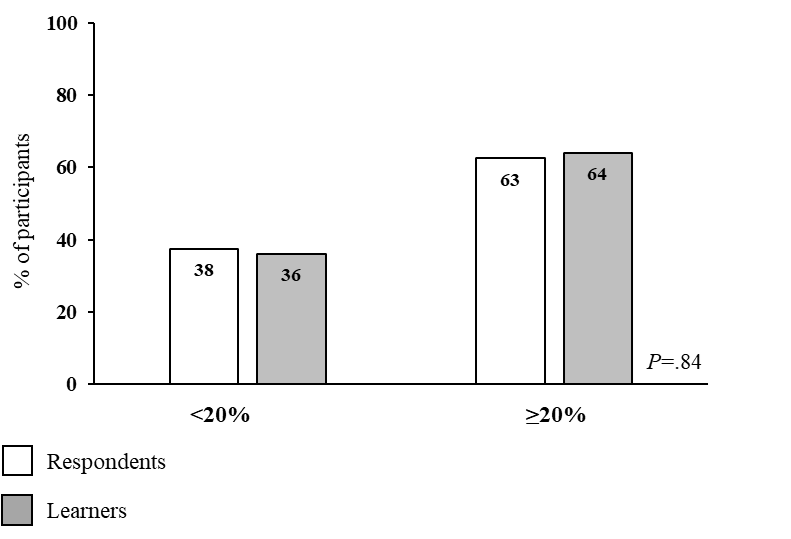
**

### Figure S2. Adjuvant or planned adjuvant therapy reported by respondents and learners in the Level 5 patient records questionnaire.

Bar graphs show the percentage of respondents (*N*=50) and learners (*N*=50) who reported adjuvant or planned adjuvant therapy for patients with (A) Ki-67 <20% or (B) Ki-67 ≥20% tumors, (C) premenopausal or (D) postmenopausal status, and germline *BRCA* wild-type status with (E) 1–3 or (F) ≥4 positive nodes. Numbers within bars indicate their value. Respondents and learners are defined as healthcare professionals who completed the pre- and post-activity questionnaires, respectively.

**(A)**

**
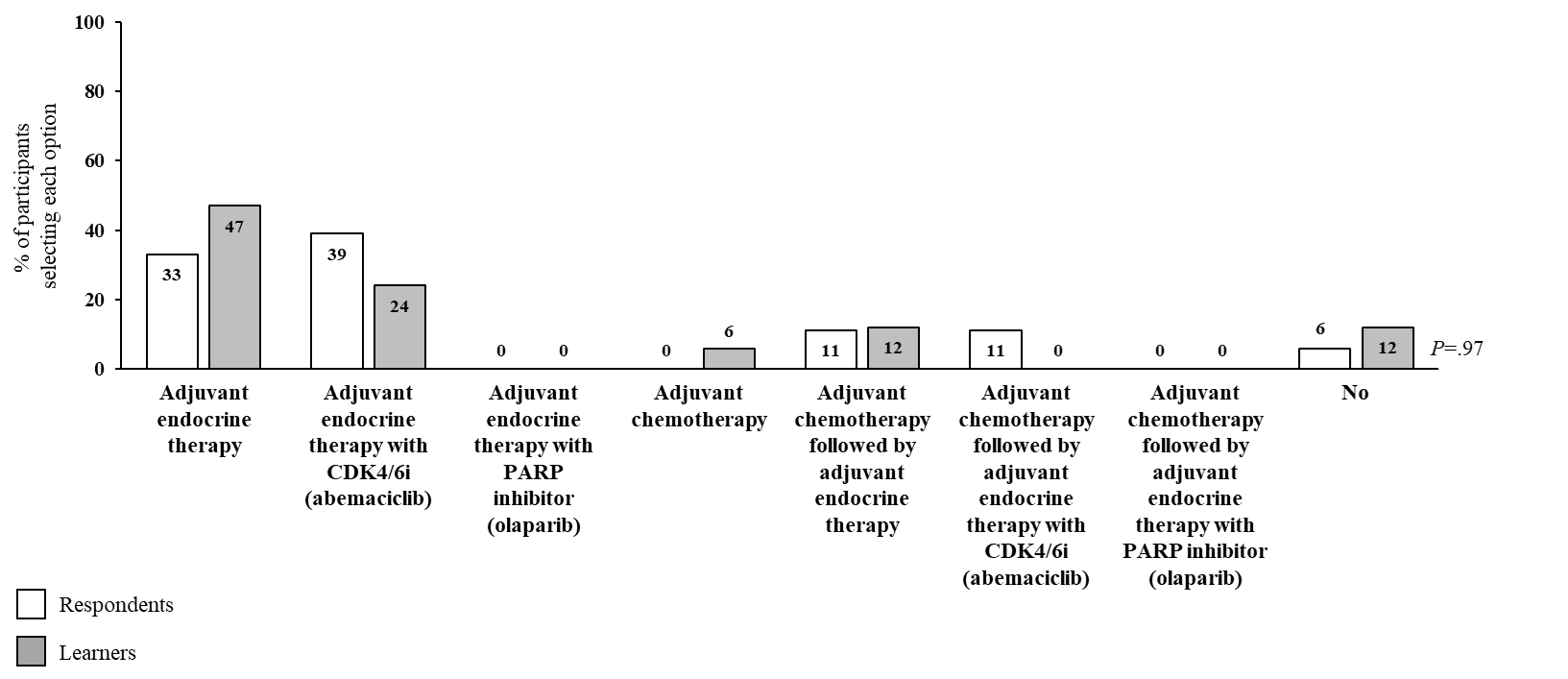
**

**(B)**

**
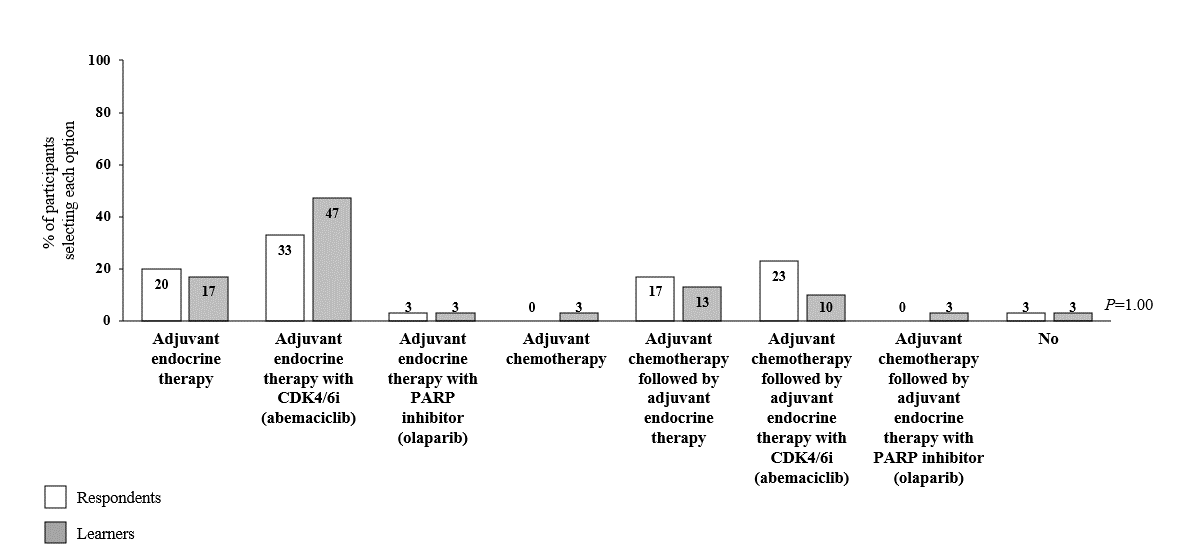
**

**(C)**

**
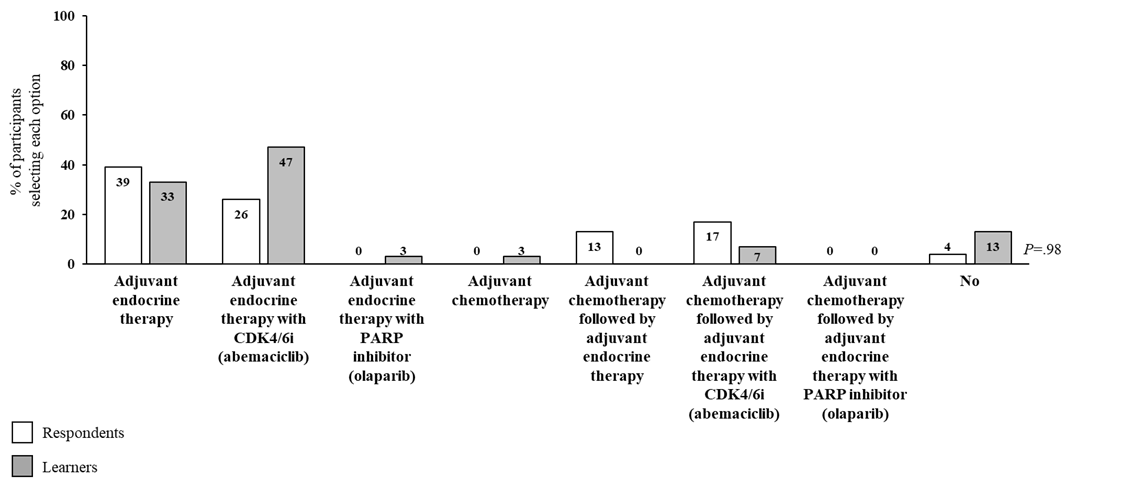
**

**(D)**

**
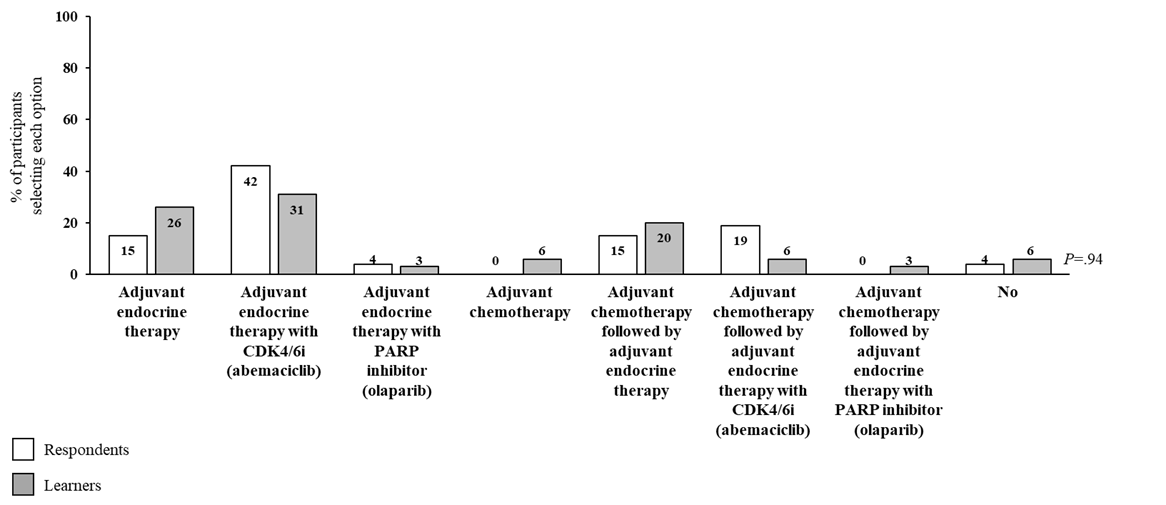
**

**(E)**

**
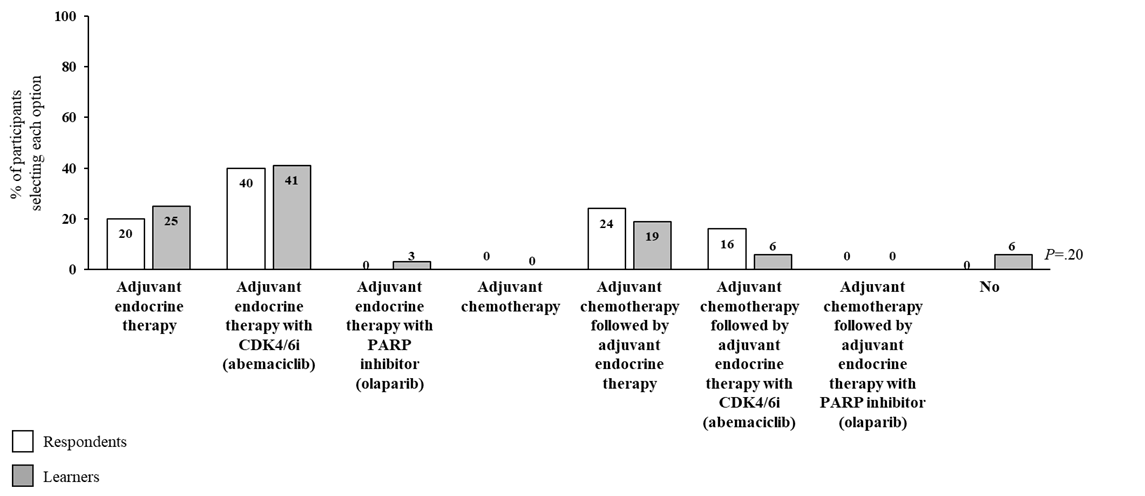
**

**(F)**

**
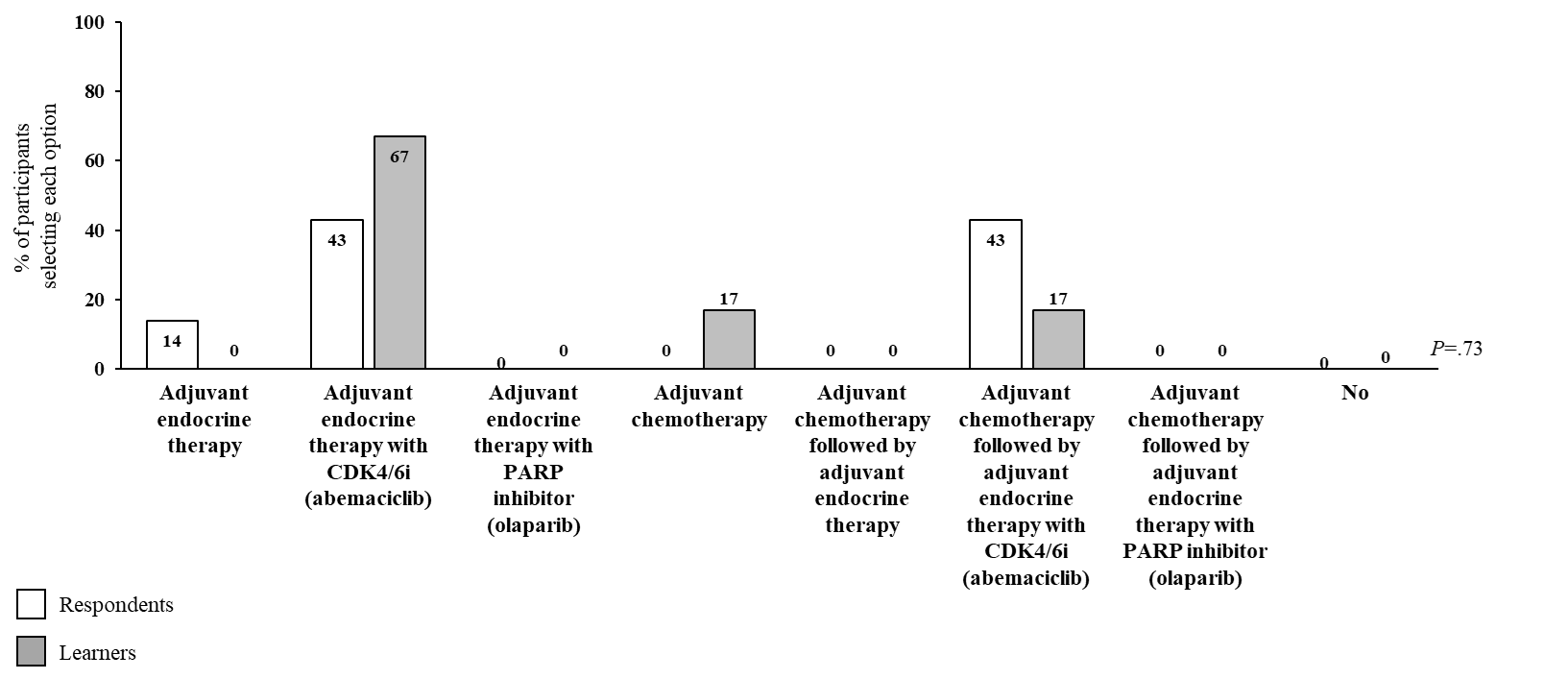
**

### Figure S3: Changes in self-reported confidence in (A) the Level 3-4 questionnaires and (B) the Level 5 questionnaires following participation in the touchMDT and touchPANEL DISCUSSION activities.

Stacked bar graphs show the percentage of respondents and learners who reported that they were not confident, a little confident, somewhat confident, moderately confident or extremely confident at treating breast cancer. Numbers within bars indicate their value. Respondents and learners are defined as healthcare professionals who completed the pre- and post-activity questionnaires, respectively.

**Abbreviation:** touchMDT, touch multidisciplinary team.

**(A)**


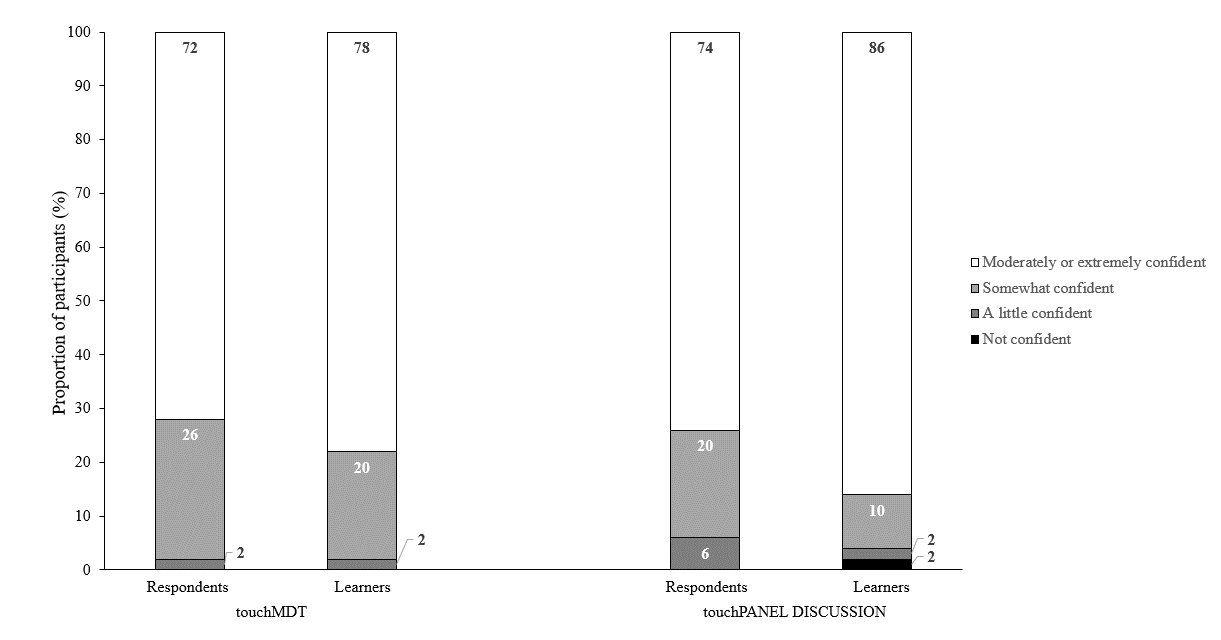


**(B)**

**
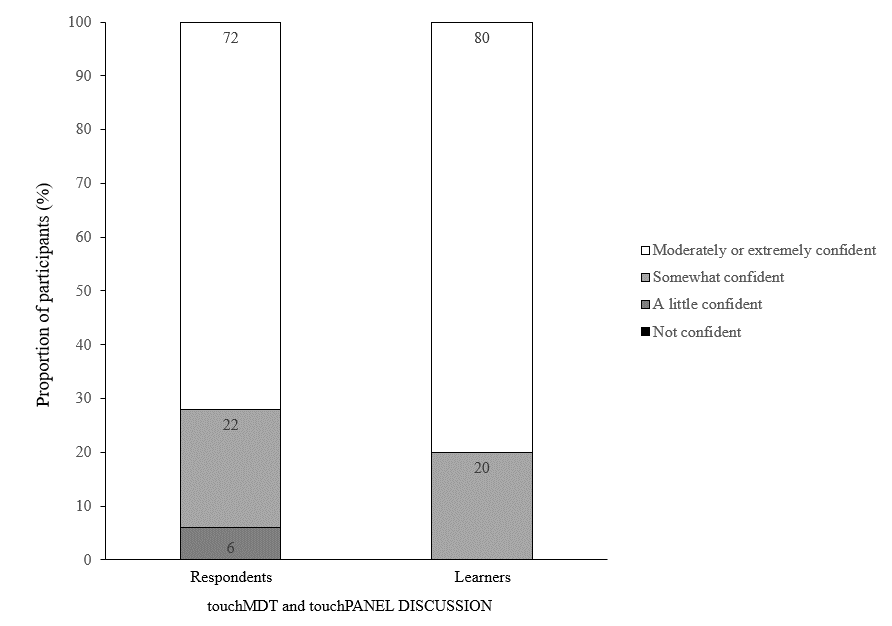
**

### Table S1: Unmet educational needs identified by the touchMDT and touchPANEL DISCUSSION learners.

| **touchMDT**  **(Level 2-4 questionnaire)** | **touchPANEL DISCUSSION (Level 2-4 questionnaire)** | **touchMDT and touchPANEL DISCUSSION**  **(Level 5 questionnaire)** |
| --- | --- | --- |
| 1. Strategies for implementing SDM in clinical practice | 1. Using genetic and biomarker testing to guide therapy choice for patients with high-risk HR+ HER2- EBC | 1. Using genetic and biomarker testing to guide therapy choice for patients with high-risk HR+, HER2- EBC |
| 2. Effective communication strategies in SDM to optimize patient outcomes | 2. Individualization of treatment choice for patients with high-risk HR+ HER2- EBC | 2. Understanding the latest data on emerging treatments for high-risk HR+ HER2- EBC |
| 3. Selecting and integrating patient decision aids to support meaningful SDM | 3. Applying the latest guideline recommendations for management of patients with high-risk HR+ HER2- EBC | 3. Applying the latest guideline recommendations for management of patients with high-risk HR+ HER2- EBC |

The top three unmet educational needs are shown, as identified by learners who completed the Level 2-4 and Level 5 questionnaires following launch of the touchMDT and touchPANEL DISCUSSION activities. Learners were required to rank four, predefined, potential educational gaps in response to the question “What do you think is the most important unmet educational need in this therapy area?”

**Abbreviations:** EBC, early breast cancer; HER2-, human epidermal growth factor receptor 2 negative; HR+, hormone receptor positive; SDM, shared decision making; touchMDT, touch multidisciplinary team.
